# RRT-Guided and Spatial Attention-Based TD3 Deep Reinforcement Learning Method for Safflower Filament Harvesting Path Planning

**DOI:** 10.3390/s26196250

**Published:** 2026-10-01

**Authors:** Bangbang Chen, Xiangdong Liu, Liqiang Wang, Baojian Ma, Kejia Zhuang

**Affiliations:** 1 School of Mechatronic Engineering, Xinjiang Institute of Technology, Aksu 843100, China; chenbangbang@st.xatu.edu.cn (B.C.); 2019049@xjit.edu.cn (L.W.); 11813019@zju.edu.cn (B.M.); 2 School of Mechanical Electronic Engineering, Wuhan University of Technology, Wuhan 430070, China; zhuangkj@whut.edu.cn

**Keywords:** safflower filaments, harvesting path, reinforcement learning, attention mechanism, gated fusion

## Abstract

Path planning for safflower filament harvesting is a critical component for ensuring the efficient operation of robotic harvesting systems. This paper proposes a path planning algorithm named SA-TD3-RRT, which integrates the Rapidly-exploring Random Tree (RRT), Twin Delayed Deep Deterministic Policy Gradient (TD3), and a spatial attention mechanism to achieve hierarchical planning of safflower filament harvesting paths. In the RRT component, techniques including adaptive step size, goal-biased sampling, and Douglas-Peucker path simplification are introduced. Specifically, the adaptive step size strategy converts the fixed step size into a dynamically adjustable extension step, effectively improving both the search real-time nature and path quality of the algorithm. The purely random sampling is replaced with goal-biased sampling, guiding the search tree growth toward the goal while suppressing redundant nodes, thereby reducing path planning time. Post-processing techniques such as Douglas-Peucker are employed to significantly reduce path complexity and improve path quality. In the reinforcement learning component, a spatial attention module is added, enabling dynamic perception of obstacle distribution. A spatial awareness term is introduced into the reward function to obtain more favorable spatial attention rewards. Additionally, the smoothed RRT path is injected into the experience replay buffer as expert demonstrations, realizing policy guidance based on imitation learning. Simulation results show that under favorable operating conditions, the proposed algorithm reduces the planned path length by 85.64%, 82.69%, and 92.55% compared to TD3, DDPG, and PPO algorithms, respectively, and shortens the path planning time by 17.68% relative to the original TD3 algorithm. In a three-dimensional environment, the planned path length is reduced by 8.29% compared to TD3, and the planning time is decreased by 81.24%, reaching 4.72 s. Although the current results are based on rigorous evaluation and demonstration in a high-fidelity simulation environment that abstracts from unstructured planting conditions, the proposed SA-TD3-RRT algorithm consistently achieved significant performance gains over the baseline. This simulation-based feasibility study provides a solid algorithmic foundation and key parameter benchmarks for future deployment on physical harvesting platforms, effectively bridging the gap between algorithmic innovation and practical agricultural robotics.

## 1. Introduction

As an economic crop valued for the integrated development and utilization of its filaments, fruits, stems, and leaves-serving as a medicinal herb, dye source, oilseed, and forage [1,2]—safflower is seeing rapid industrial and commercial growth, playing an invaluable role in regional socio-economic development. In Xinjiang, China, the safflower cultivation area reaches 40,000 hectares, with an annual output of approximately 4000 tons [3]. However, safflower filament harvesting is still largely performed manually, which results in high labor intensity, low efficiency, and high costs. In recent years, with advances in artificial intelligence, agricultural robotics has provided new solutions for safflower filament harvesting [4]. Because safflower filaments and flower bulbs are close to each other and have different maturation and harvest times, adjacent fruits may act as obstacles during robotic harvesting, potentially causing collisions between the manipulator and the bulbs. Therefore, to achieve efficient, low-loss harvesting, designing an effective and rational harvesting path planning method is essential.

Currently, path planning algorithms fall into three categories: traditional algorithms, intelligent bionic algorithms, and reinforcement learning-based algorithms. Traditional algorithms include A* [5], RRT [6], and RRT-connect [7]; intelligent bionic algorithms include ant colony optimization [8] and genetic algorithms [9]. Both categories have limitations. Traditional algorithms separate perception from decision-making, making them difficult to deploy in non-stationary environments and resulting in slow path planning in complex environments. Intelligent bionic algorithms offer strong global search capability and adaptability, but they are prone to local optima and may fail to find the global optimum; additionally, their computational overhead is high and real-time performance is insufficient. The random exploration in these methods may also prevent them from finding more optimal solutions. Reinforcement learning (RL) [10] does not require prior knowledge; it can interact with the environment through trial and error to obtain feedback, optimize route selection strategies, and support autonomous and online learning. As a result, RL has been widely applied in path planning.

Current deep reinforcement learning (DRL) algorithms still face challenges such as long training times and low training efficiency. To address training efficiency, Li et al. [11] designed a novel double deep Q-network to mitigate issues of excessive randomness in action selection and slow convergence during training. They introduced a new temporal input structure to capture richer semantic information for dynamic scene path planning, along with a new experience allocation strategy that improves training efficiency, achieving a 50% reduction in training time and a 9.6% increase in success rate. Feng et al. [12] integrated deep learning into the Q-learning framework to solve the dimensionality problem in complex environments, using a correction function to improve the Q-network’s evaluation function and reduce overestimation in deep Q-networks; this resulted in 60% faster convergence and a 25% reduction in average steps. To address low efficiency and high cost in lotus seed head harvesting, Zheng et al. [13] proposed an efficient pointer network model that quickly and accurately computes harvesting paths. For 20, 40, and 80 target points, the solution time dropped to below 0.02 s, and path lengths remained within 10 units; the model maintained superior performance even when deployed on an NVIDIA Jetson Nano. Kumaa et al. [14] built upon Q-networks by using topological graphs of the environment to learn initial paths and proposed a β-decay transfer learning algorithm to train agents in new environments, achieving over 98% accuracy in learning all paths.

The current trend in path planning is to combine reinforcement learning algorithms with traditional path planning algorithms. Traditional algorithms possess strong sampling and search capabilities and can provide reference paths or intermediate waypoints for DRL. At the same time, DRL performs point-to-point tasks between node pairs, enabling agents to obtain smoother paths [15]. Sampling-based path planning methods do not require reward functions in complex environments and achieve higher success rates than RL, making them suitable for providing successful experience references for RL. RL, in turn, leverages those experiences to enhance its exploration capability and ultimately learns an effective path planning policy. Gao et al. [16] combined TD3 with PRM: PRM provides global intermediate goals, while TD3 handles local path planning, improving model generalization in large scenes. Li et al. [17] proposed a hybrid method combining a traditional path planner with DRL to obtain paths in Cartesian space. Florensa et al. [18] introduced an inverse curriculum generation method that requires neither expert demonstrations nor manually designed reward functions. Chiang et al. [19] proposed a kinematic dynamic planning framework combining DRL with sampling-based planning methods: DRL learns an obstacle-avoidance policy as a local planner, and a reachability estimator guides RRT growth, enabling motion planning without an optimal steering function. Cai et al. [20] combined the experience replay mechanism of DRL with RRT* to propose a new algorithm that compensates for the insufficient exploration of TD3; path lengths in both 2D and 3D environments were reduced by more than 10%. Many researchers have conducted in-depth studies on task allocation and path planning, proposing various methods to optimize operational efficiency and quality. Recently, Hazem et al. [21] proposed a fuzzy-TD3 hybrid framework for trajectory tracking of a robotic manipulator, demonstrating the potential of integrating fuzzy logic with TD3 for improved robustness in dynamic environments. However, given the complex, unstructured growth environment of safflower-such as uneven crop distribution-existing algorithms struggle to balance the real-time nature and accuracy of path-planning tasks.

To address this gap, this paper proposes a hierarchical planning algorithm that integrates RRT with reinforcement learning, named SA-TD3-RRT. Its main innovations are as follows:

(1) A goal-biased dynamic decay strategy is introduced into the traditional RRT algorithm to guide the search tree toward the goal and reduce redundant nodes. An adaptive step-size mechanism is designed to further improve search efficiency. A three-stage path post-processing pipeline-combining Douglas-Peucker simplification, Shortcut straightening, and wandering detection-achieves near-optimal path length while ensuring path feasibility.
(2) A spatial attention-gated fusion module is built upon the TD3 algorithm. A multi-head self-attention mechanism captures global obstacle distribution relationships and extracts local geometric features, while a gating mechanism adaptively fuses the two types of features. A spatial awareness term is added to the reward function, guiding the model to focus on key environmental features and further improving obstacle-avoidance capability and path smoothness.
(3) An expert guidance training mechanism based on imitation learning is proposed. Smooth paths generated by the improved RRT are converted into state-action pairs and stored in the experience replay buffer. By mixing ordinary samples with expert samples and designing a critic expert guidance loss, the algorithm effectively addresses the blind exploration and slow convergence typically observed in early reinforcement learning training, substantially reducing the policy’s exploration space.

## 2. Materials and Methods

### 2.1. Problem Description and Physical Constraints

The mechanical harvesting of safflower filaments is an operational task that demands high precision and safety. Unlike conventional industrial grasping, safflower plants exhibit unstructured, densely distributed growth characteristics, with flower bulbs and filaments spatially interleaved and asynchronous maturation periods, imposing numerous physical constraints on the harvesting manipulator during operation. This section systematically elaborates on these constraints from three perspectives: the harvesting workflow, safety requirements, and workspace characteristics. On this basis, a rigorous mathematical formulation of the path planning optimization problem is established.

#### 2.1.1. Sequential Requirements of the Harvesting Workflow

Safflower filament harvesting must adhere to a precise temporal action sequence to avoid damaging flower bulbs, breaking stems, or missing effective fibers. The harvesting workflow comprises the following five stages:

(1) Target identification and localization: The scene is perceived using visual sensors, and the position and orientation of safflower filaments are identified through object detection algorithms, with their three-dimensional world coordinates computed.
(2) Approach to target: The end-effector of the manipulator moves from its current position toward the target filament, traveling along a collision-free path at high speed while maintaining a safe distance from surrounding targets.
(3) Alignment and descent: The end-effector descends vertically to a position directly above the target filament and fine-tunes its orientation to align with the picking point. During this process, the descent speed must be strictly controlled to avoid crushing petals or dislodging immature flower bulbs.
(4) Grasping and picking: The actuator performs the picking action, which involves multiple control operations; however, at the path planning stage, it is only required to ensure that the end-effector reaches the precise picking pose.
(5) Lifting and transfer: After picking, the end-effector rises to a safe height. Throughout the entire process, the end-effector must avoid previously picked areas and other plants.

The above workflow indicates that path planning must not only provide a geometric route from the start point to the goal but also accommodate vertical motion modes such as lifting, horizontal movement, and descent, ensuring that obstacle avoidance and precision requirements are met at each operational stage.

#### 2.1.2. Safety Distance and Obstacle Avoidance Constraints

The spatial gaps between safflower plants are narrow; the diameters of adjacent flower bulbs are approximately 20–40 mm, and the inter-stem spacing is often less than 50 mm. To prevent physical contact between the manipulator and flower bulbs, path planning must enforce safety distance constraints. The distance constraints consist of two parts:

Minimum approach distance: The Euclidean distance between the end-effector and any obstacle must be greater than a preset safety margin. In this paper, the safety margin is set to 30 mm for 2D planning and 15 mm for 3D planning.

Safe height: During horizontal movement, the end-effector must remain above a safe height to avoid colliding with low-lying flower bulbs. Based on the actual workspace (i.e., the workspace of the manipulator used in this paper), the safe height is set to 1000 mm. All horizontal path segments are executed at this height layer, and vertical movements are performed only for ascending and descending above the target points, thereby decoupling the 3D obstacle avoidance problem into horizontal-layer planning and vertical lifting/lowering planning.

#### 2.1.3. Mathematical Formulation of the Path Planning Optimization Problem

Based on the physical constraints described above, safflower filament harvesting path planning can be formalized as a constrained optimization problem. Let the discrete path of the manipulator end-effector in three-dimensional space be an ordered set of points *P* = {*p*_0_, *p*_1_, …, *p_N_*}, where *p_i_* is the three-dimensional coordinate of the *i*-th point, *p*_0_ is the start point, and *p_N_* is the final goal point. The objective function is defined as the weighted total cost of the path:

(1) $$J(P)=\lambda_{1}\sum_{i=0}^{N-1}\left\|p_{i+1}-\left.p_{i}\right\|\right.+\lambda_{2}\sum_{i=1}^{N-1}\kappa (p_{i-1},p_{i},p_{i+1})+\lambda_{3}N$$

where the first term is the Euclidean path length, the second term is the path curvature penalty, and the third term is a regularization term on the number of path points. The coefficients *λ*_1_, *λ*_2_, *λ*_3_ are weight factors used to balance efficiency, smoothness, and computational complexity. The curvature κ at point pi is approximated by the angle between the two consecutive edge vectors; the curvature function κ is defined as:

(2) $$\kappa (p_{i-1},p_{i},p_{i+1})=\frac{2{\left\|\left(p_{i}-p_{i-1})\times (p_{i+1}-p_{i}\right)\right\|}_{2}}{{\left\|\left(p_{i}-p_{i-1}\right)\right\|}_{2}{\left\|p_{i+1}-p_{i}\right\|}_{2}({\left\|p_{i}-p_{i-1}\right\|}_{2}+{\left\|p_{i+1}-p_{i}\right\|}_{2})}$$

This optimization problem is non-convex, high-dimensional, and subject to complex constraints, making it difficult to solve using conventional analytical methods. Therefore, this paper adopts a hierarchical solution strategy: the upper layer employs an improved RRT to generate feasible paths as expert demonstrations, the lower layer uses a spatial attention-enhanced TD3 algorithm for local refinement, and an approximate optimal solution is finally obtained through post-processing.

#### 2.1.4. Obstacle Space Modeling

In the manipulator harvesting scenario of this paper, obstacles are uniformly modeled as bounding boxes. A 2D obstacle is defined as O_2*D*_ = {(*x*, *y*, *w*, *h*)}, and a 3D obstacle is defined as O_3*D*_ = {(*x*, *y*, *z*, *w*, *h*, *d*)}. To simulate the unstructured, densely distributed growth environment of safflower plants, this paper adopts a heuristic generation strategy based on object detection results: obstacles are generated with random offsets centered at the detected true targets. This approach produces more complete obstacle configurations for both sparse-target and multi-target complex environments. The generated obstacles are used to simulate the dense, unstructured distribution of safflower plants, though real three-dimensional scan geometries are not employed.

### 2.2. SA-TD3-RRT: A Hybrid RRT and TD3 Path Planning Algorithm for Safflower Filament Harvesting

TD3 employs a twin-Q-network architecture that takes the minimum value to suppress overestimation, delays policy updates to reduce error accumulation, and applies target policy smoothing to enhance robustness. At the same time, it allows flexible integration of expert demonstrations, and its deterministic action outputs ensure high repeatability of end-effector poses. In terms of overestimation suppression, sample efficiency, and precise localization, TD3 is better suited to meet the requirements of safe obstacle avoidance and accurate positioning in safflower filament harvesting. In contrast, although DDPG has a structurally concise design, its single-Q-network architecture leads to overestimation issues that can easily cause collisions in dense obstacle environments. PPO, while effective, cannot directly leverage the offline expert demonstrations generated by RRT for experience reuse. Although SAC encourages exploration through entropy regularization, its stochastic policy outputs produce action distributions that require additional sampling, which introduces unnecessary jitter in scenarios where the end-effector must precisely reach the picking point. Therefore, this paper selects TD3 as the baseline algorithm.

**Figure 1:**
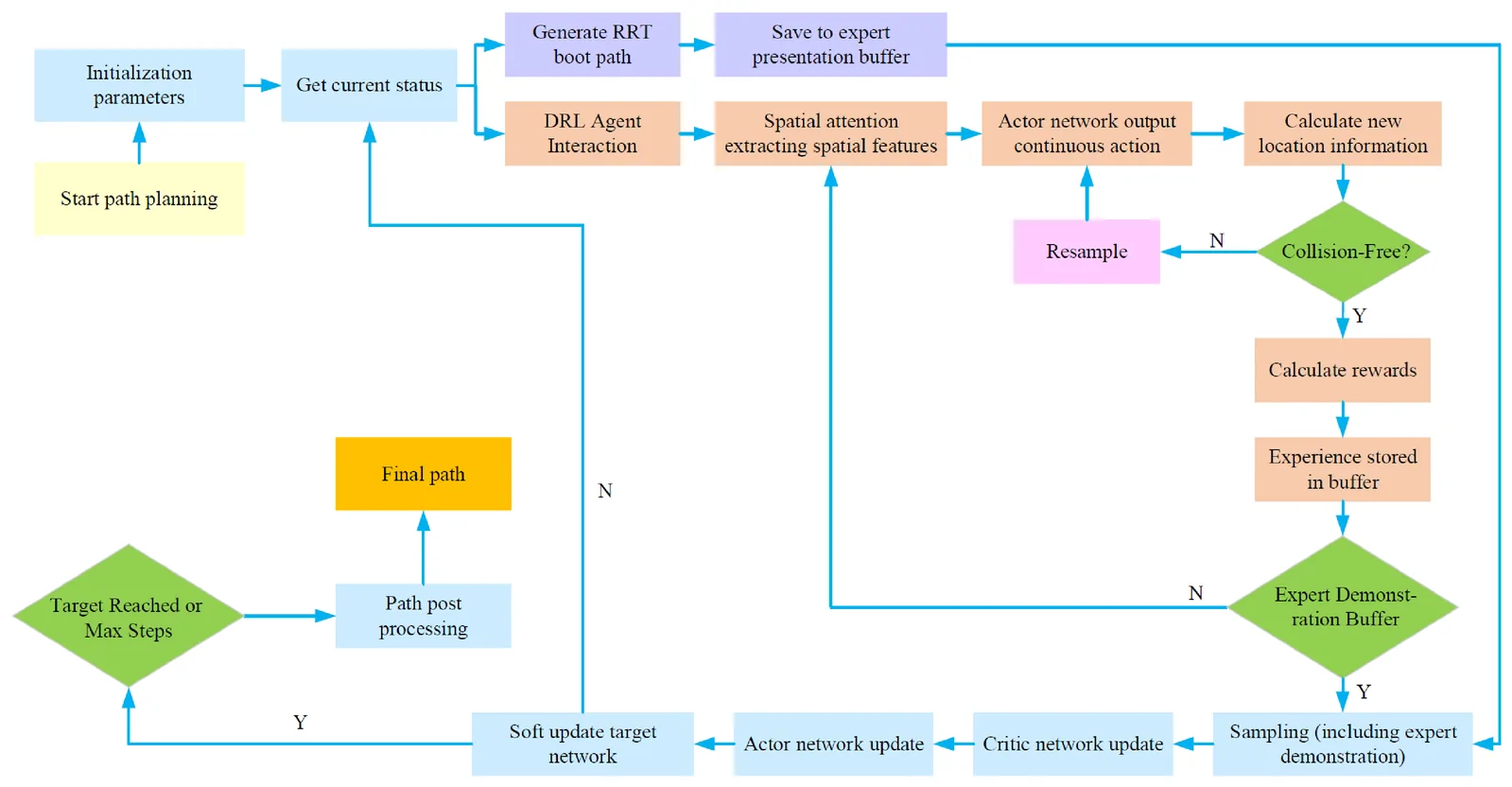
SA-TD3-RRT planning strategy diagram.

In the complex natural environment where safflower grows, the flowering period, plant height, and spatial distribution of safflower flowers and fruits vary with changes in field agronomic practices. Existing safflower filament harvesting robots often suffer from low efficiency and poor performance due to suboptimal harvesting sequence planning. To address this issue, this paper further improves the RRT and TD3 algorithms to redesign a path planning algorithm for safflower harvesting robots in complex environments, named SA-TD3-RRT. The planning strategy is illustrated in Figure 1. First, the improved RRT generates expert paths and stores them in the experience replay buffer as guidance. Second, the spatial attention module encodes the relative relationship between the agent’s own state and obstacles into spatial features. The Actor network outputs continuous actions accordingly, which are discretized and executed as displacements. At each step, a reward is calculated, and the experience is stored in the buffer. When sufficient samples are available, data containing expert demonstrations are sampled from the buffer. The twin Critic networks minimize the TD error, while the Actor network updates its policy by maximizing Q-values and imitating expert actions. Upon reaching the goal, the path undergoes a series of post-processing steps, finally outputting a smooth, safe, and efficient path. To clearly present the logical relationships and execution sequence of each module, Algorithm 1 provides the detailed pseudocode of the algorithm.
**Algorithm 1.** SA-TD3-RRT pseudocodeInput: S, G, O, B Output: P
  1:  n ← S, P ← [n], cf ← 0
  2:  for i = 1 to I do
  3:     S_own_, S_int_, mask ← GetState (n, G, O)
  4:     a_cont_ ← Actor (S_own_, S_int_, mask); a_disc_ ← Discretize (a_cont_)
  5:     n′ ← Apply (n, a_disc_, δ, B)
  6:     if CollisionFree (n′, O) then
  7:       S′_own_, S′_int_, mask′ ← GetState (n′, G, O)
  8:       r ← Reward (S_own_, a_cont_, S′_own_, Done (n′, G))
  9:       Buffer. Add (S_own_, S_int_, mask, a_cont_, r, S′_own_, S′_int_, mask′)
10:       P ← P ∪ {n′}, n ← n′, cf ← 0
11:       if Done (n′, G) then break
12:     else
13:       cf ← cf + 1; if cf > C then Backtrack (n, P) end if
14:     end if
15:     if i mod F = 0 and |Buffer| > B_size then UpdateNets () end if
16:  end for
17:  P ← PostProcess (P, attention_history)
18:  return P

Table 1 provides the definitions and descriptions of the key variables and functions employed in the code.

### 2.3. Design of the RRT-Guided Safflower Filament Harvesting Trajectory Planning Algorithm

**Figure 2:**
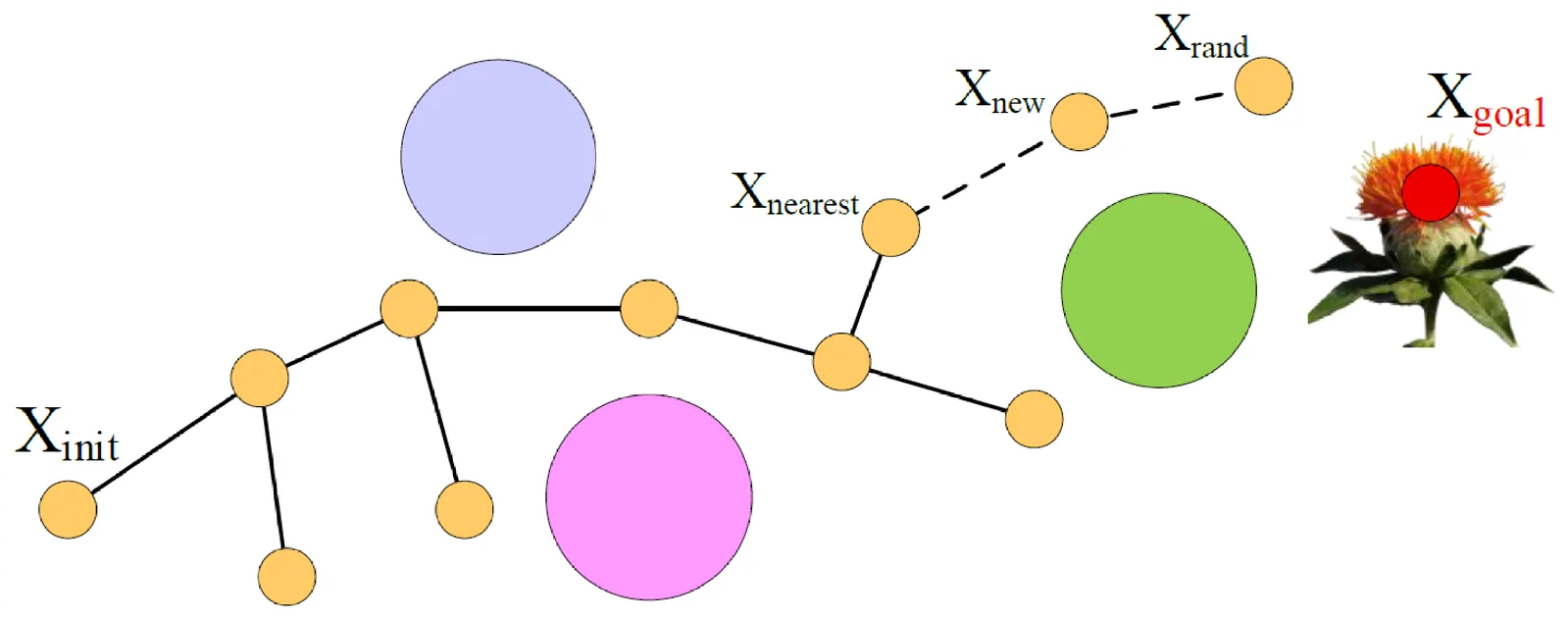
Original RRT algorithm search logic diagram.

**Figure 3:**
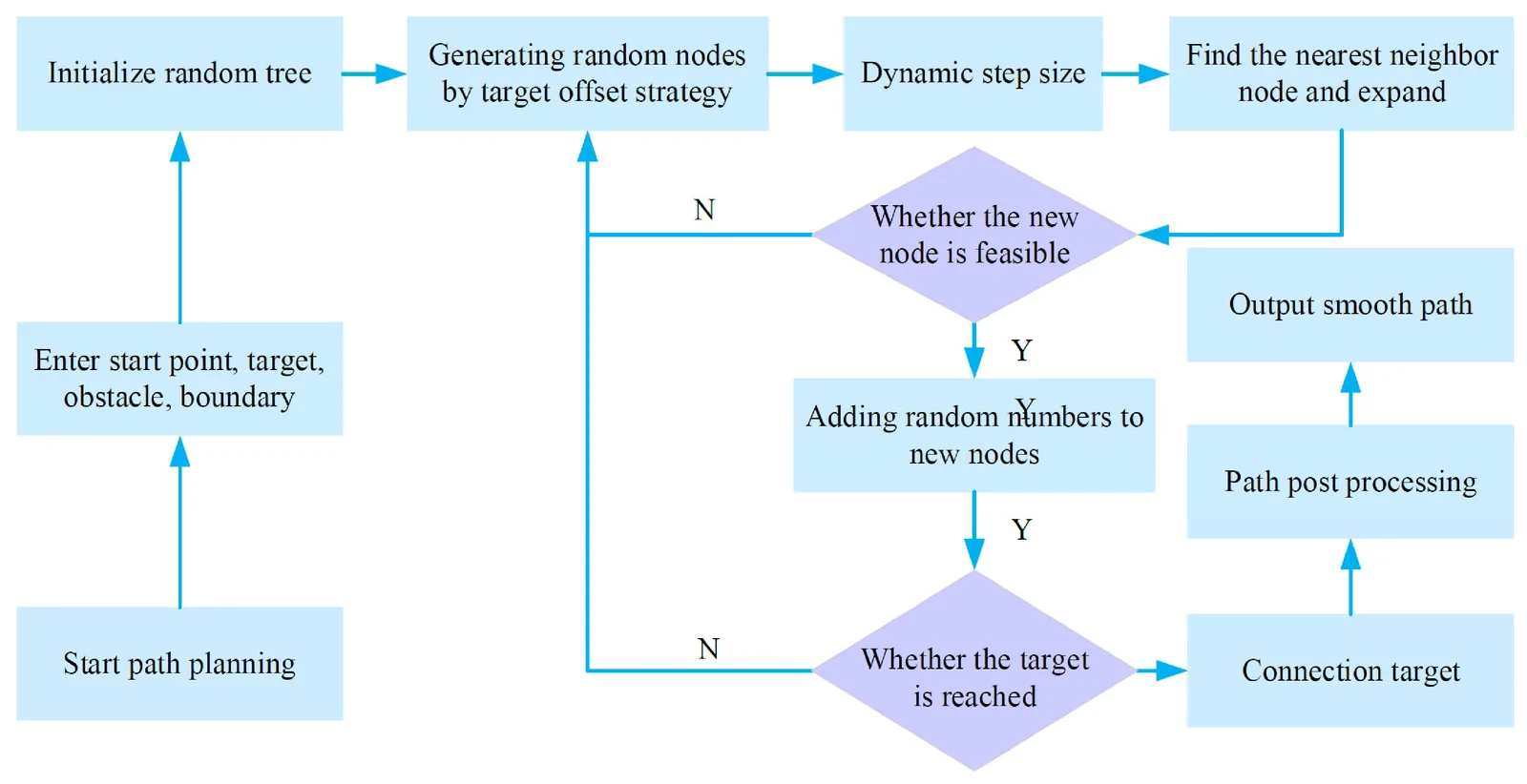
Flowchart of the improved RRT path planning algorithm.

In this paper, the RRT algorithm is used as the demonstration expert for the deep reinforcement learning algorithm. As shown in Figure 2, the original RRT algorithm suffers from a high proportion of invalid samples for target search, excessive redundant turning points in the generated path, excessive path length, and poor smoothness. To address these issues, a goal-biased strategy [22], an adaptive step-size strategy [23], and path smoothing optimization are incorporated into RRT. The improved RRT algorithm workflow is shown in Figure 3.

#### 2.3.1. Optimizing RRT with a Goal-Biased Strategy

**Figure 4:**
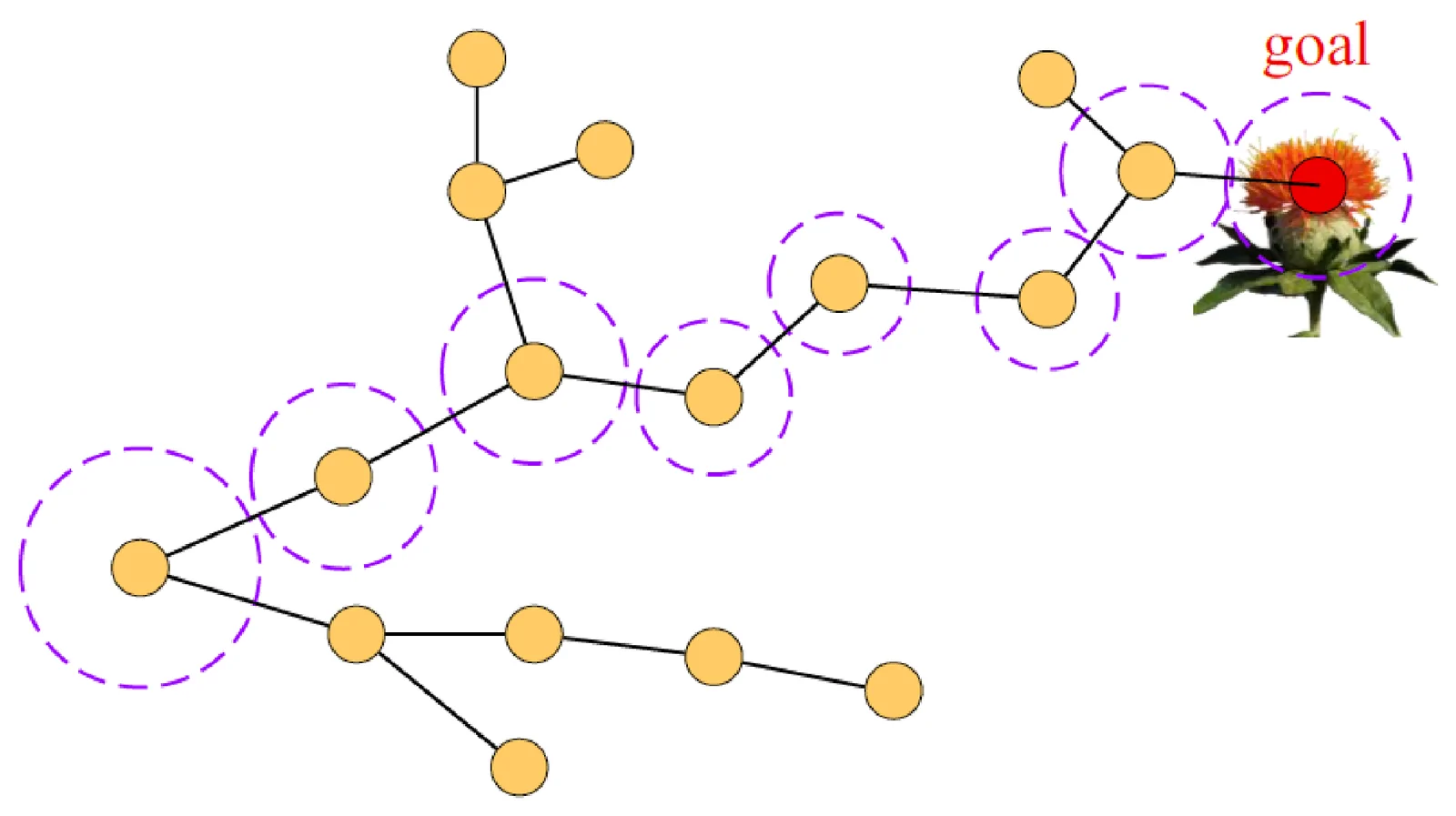
Schematic diagram of the goal-biased strategy.

The traditional RRT algorithm uses uniform random sampling in space. While this sampling method achieves probabilistic completeness, it lacks direct guidance toward the safflower harvesting target, generating many redundant nodes and increasing path planning time during harvesting. To overcome this, this paper adopts a goal-biased strategy to limit the number of nodes in the search tree and guide the search toward the harvesting goal point, thereby reducing path planning time. As illustrated in Figure 4, the goal-biased strategy takes the goal point as the random sampling point with a certain probability, so that new nodes have a certain probability of growing toward the goal point, enhancing goal-directed behavior during path search.

In the goal-biased sampling process, a goal bias probability P is set. A uniformly distributed random probability value P_1_ is generated. If P_1_ < r, the algorithm enters the goal-directed phase, where the goal point is used as the sampling point, and the new node grows in the direction of the goal. If r < P_1_ ≤ P, the goal-biased sampling phase begins: a random sampling function generates a sampling point *x*_sample_ within the sampling space. The sampling principle is expressed in Equations (3)–(5):

(3) $$x_{\mathrm{rand}}=\left\{\begin{array}{c} x_{\mathrm{goal}}{,\;P}_{1}<r\\ x_{\mathrm{sample}}{,\;r<P}_{1}\leq P \end{array}\right.$$

*x* = *x*_nearest_ + *λ* × (*x*_goal_ − *x*_nearest_) (4)

*y* = *y*_nearest_ + *λ* × (*y*_goal_ − *y*_nearest_) (5)

In the equations, *λ* is the step-size coefficient, P is the goal sampling rate, *x*_sample_ represents a random sampling point within the sampling space, *x*_goal_ denotes the path goal point, *x*_rand_ represents the sampling point obtained after goal-biased sampling, *x* and *y* are the coordinates of the new sampling point, *x*_nearest_ and *y*_nearest_ are the coordinates of the nearest node, and r is the goal sampling rate, which is set to 0.1 in this paper.

At the same time, the concept of a dynamic step size is incorporated to allow the bias probability to decay dynamically, further accelerating the convergence speed. The decay formula is given in Equation (6):

(6) $$P(i)=P_{\mathrm{basic}}*\alpha^{\frac{i}{i_{\max}}*k}$$

where *P*_basic_ is the initial bias probability, set to 0.3 in this paper; *α* is the decay coefficient, set to 0.99; *i* is the current iteration number; *i*_max_ is the maximum number of iterations; and k is the factor regulating the decay speed; a larger k leads to faster decay of the bias probability.

#### 2.3.2. Optimizing RRT with an Adaptive Step-Size Strategy

**Figure 5:**
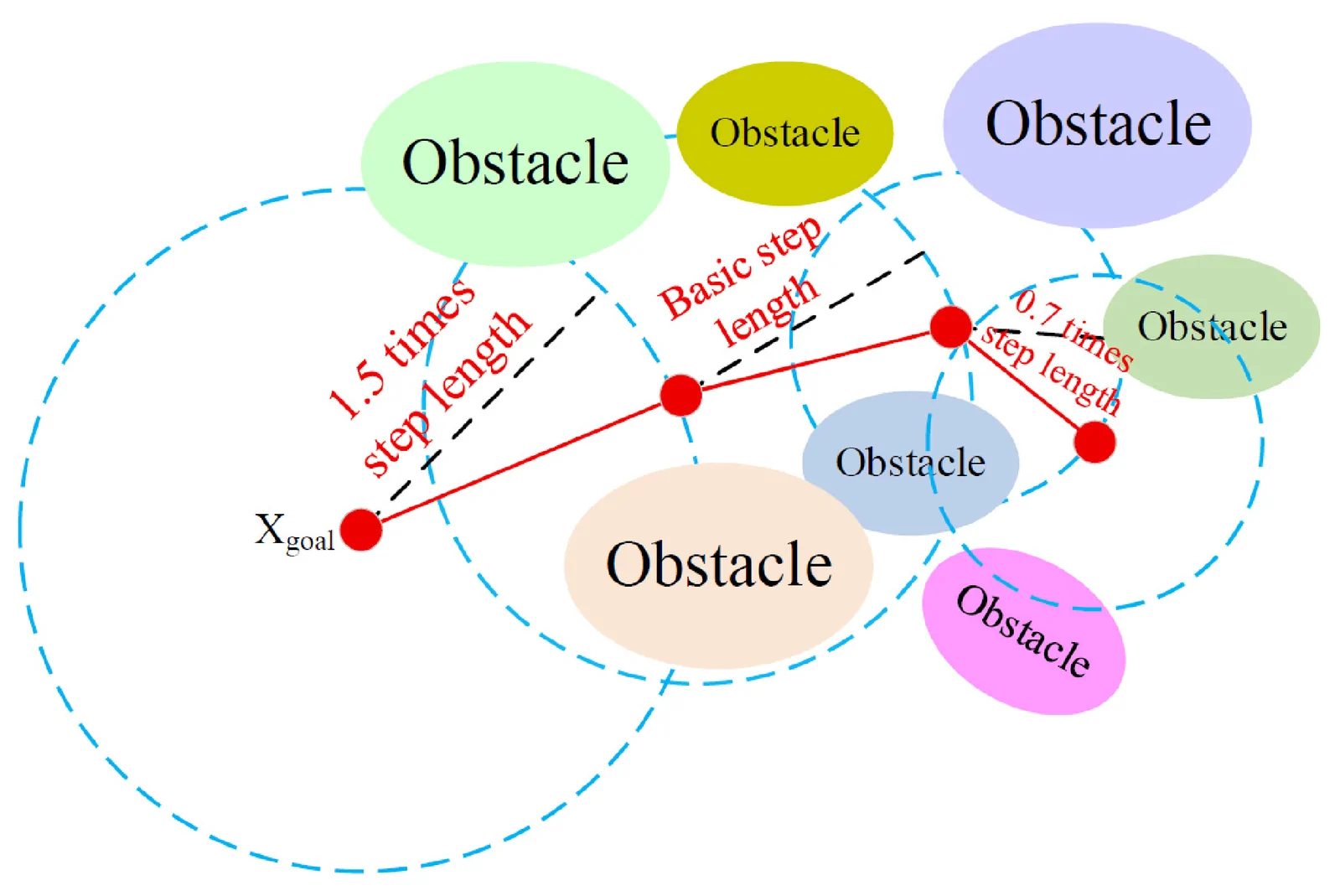
Schematic diagram of the dynamic step-size strategy.

When using the RRT algorithm for path planning, the growth step size of the random tree is fixed. An excessively large or small step size can impair search efficiency, leading to slow convergence in open areas and failure to expand in complex environments, thereby severely affecting path planning efficiency. To address these issues, this paper introduces an adaptive step-size strategy, as illustrated in Figure 5. The step size is dynamically adjusted according to the ratio of the current iteration number to the total iteration number, enabling the algorithm to achieve “fast exploration in the early stage and fine convergence in the later stage,” thus improving both search real-time nature and path quality.

Guided by the adaptive step-size strategy, the expansion step size varies with the current iteration number. The specific process is as follows: (1) At the beginning of the iteration, the step size is expanded to 1.5 times the base step size for rapid space exploration, so as to find a feasible path as quickly as possible. (2) When the iteration count is between 1/3 and 7/10 of the total number of iterations, the step size is linearly reduced from the base step size to 0.7 times the base step size, thereby balancing exploration and accuracy, avoiding premature local convergence, and gradually refining the path. (3) During the final 1/3 of iterations, the step size is maintained at 0.7 times the base step size to improve path accuracy, avoid collisions with obstacles, and optimize path quality.

The linear decay formula is shown in Equation (7):

(7) $$S_{i}=l_{\mathrm{base}}\times \left[\alpha_{\mathrm{step}}-(\alpha_{\mathrm{step}}-\beta_{\mathrm{step}})\times \frac{i}{i_{\max}}\right]$$

where S*_i_* represents the step size at the corresponding iteration number; *l*_base_ is the base step size; *α_step_* is the initial amplification factor; *β_step_* is the final reduction factor; *i* is the current iteration number; *i*_max_ is the maximum number of iterations. In this paper, *l_base_* is set to 0.5, *α* is set to 1.5, and *β* is set to 0.5.

#### 2.3.3. Path Smoothing Optimization for RRT

This algorithm combines the Douglas-Peucker (DP) simplification algorithm [24] and the Shortcut algorithm [25] to simplify and optimize the planned path. The initial path output by the original planner typically contains redundant collinear points, jagged turns, and large-scale detours. The post-processing module sequentially calls three sub-algorithms according to a fixed procedure, as shown in Equation (8):

Path_final_ = WanderingDetection(Shortcut(DP(Path_initial_, *ε*), *T_sc_*), *ρ*) (8)

where *ε* is the DP threshold, *T_sc_* is the number of Shortcut iterations, and *ρ* is the wandering path length ratio threshold. In this paper, *ε* is set to 0.05, *T_sc_* is set to 500, and *ρ* is set to 0.3.

The DP algorithm recursively extracts the “key turning points” of the path. Its core is the perpendicular distance *d* from a point to the line connecting the start and end points, calculated as shown in Equation (9):

(9) $$d(P_{i},P_{s},P_{e})=\frac{\left|(y_{e}-y_{s})x_{i}-(x_{e}-x_{s})y_{i}+x_{e}y_{s}-y_{e}x_{s}\right|}{\sqrt{{\left(y_{e}-y_{s}\right)}^{2}+{\left(x_{e}-x_{s}\right)}^{2}}}$$

where P*_i_* is the current path point under inspection, P*_s_* is the start point of the current recursive segment, P*_e_* is the end point of the current recursive segment, *x_i_*, *x_s_*, *x_e_* are the *x-coordinates* of P*_i_*, P*_s_* and P*_e_* respectively, and *y_i_*, *y_s_*, *y_e_* are the corresponding y-coordinates.

If the maximum distance *d*_max_ > *ε*, the segment is split at that point, and both sub-segments are simplified recursively; otherwise, the entire segment is approximated as a straight line edge. DP operates globally: it removes dense intermediate points introduced by noise or random sampling along straight-line travel while strictly preserving geometric constraints at large-angle turns. After DP processing, the number of path points is significantly reduced, but key bends are fully retained, providing a concise initial skeleton for subsequent optimization.

The Shortcut algorithm runs on the DP-simplified path *P*_dp_. It attempts to directly connect two points by random sampling to bypass redundant turning points. In each iteration, two indices *i* < *j* are randomly selected. If the edge (*P_i_*, *P_j_*) passes collision detection, all intermediate nodes are removed.

This algorithm primarily handles non-collinear but actually straighten-able regions that DP cannot resolve. Through a large number of random attempts, it can delete these implicitly redundant segments with very high probability. Because DP has already substantially reduced the number of path points, the search space for the Shortcut algorithm is diminished, greatly increasing the probability of successful connections. The combination of DP and Shortcut balances path length and validity.

Although the combination of the two methods described above optimizes most redundant paths, when the path contains large detours or regular wandering, the DP and Shortcut algorithms cannot accurately eliminate the redundancy. In such cases, a new method is needed for global correction to ensure path correctness. The main calculation formula is shown in Equation (10):

(10) $$\eta =\frac{L_{a}}{L_{s}},\;L_{a}=\sum_{k=0}^{N-1}\left|\left|P_{k+1}-P_{k}\right|\right|$$

where *η* is the path efficiency ratio, *L_a_* is the actual path length, *L_s_* is the straight-line distance from the start point to the goal point, *N* is the total number of path points, and ||*P_k_*_+1_ − *P_k_*|| is the Euclidean distance between two adjacent points.

If *η* > 1 + *ρ*, the path is judged to contain a detour. In this case, the DP algorithm is re-executed with a new threshold *ε*′ = 0.1 *L_s_* to remove intermediate loop-closure points. Throughout the path smoothing optimization process, DP and Shortcut are applied in a forward-passing manner to gradually optimize the path. When the optimization effect is insufficient, wandering detection performs a fallback processing step. Finally, a shortened feasible path is obtained while maintaining collision-free properties.

### 2.4. Real-Time Path Planning for Safflower Filament Harvesting Based on an Improved TD3 Reinforcement Learning Algorithm

In complex scenarios, the state space of the agent in reinforcement learning typically includes its own position, the goal point position, and the geometric attributes of multiple obstacles. When the number of obstacles is variable, efficiently encoding the dynamic relationships among multiple entities becomes a bottleneck. The multi-head self-attention mechanism in Transformers can automatically learn pairwise dependencies among sequence elements. However, a pure attention mechanism lacks explicit spatial position priors and local geometric awareness, which may lead to the neglect of critical information such as relative distances and obstacle shapes. To address this issue, this paper proposes an enhanced attention module that integrates spatial grid convolution features along with mechanisms such as spatial awareness rewards, thereby constructing a spatial attention-enhanced path planner.

#### 2.4.1. TD3 Reinforcement Learning Algorithm

Reinforcement learning is typically described through a Markov decision process, which can be represented by the tuple *M* = (*S*, *A*, *r*, *p*, *γ*), where *S* and *A* denote the set of states and actions, respectively; *p* is the probability of transitioning from the current state to the next state; *r* is the reward obtained from the environment for a state transition; and *γ* ∈ (0,1) is the discount factor that determines the priority of rewards. At time step *t*, given a state *s* ∈ *S*, the agent selects an action *a* ∈ *A* according to a policy *u*, transitions to the next state *s_t_*_+1_ ∈ *S*, and receives a reward *r*. The agent aims to maximize the discounted return $R_{t}=\sum_{i=t}^{T}\gamma^{i-t}r(s_{i},a_{i})$, which can be measured by the value function *Q* as shown in Equation (11):

*Q^μ^*(*s*, *a*) = *E_μ_*[*R_t_*|*s*, *a*] (11)

**Figure 6:**
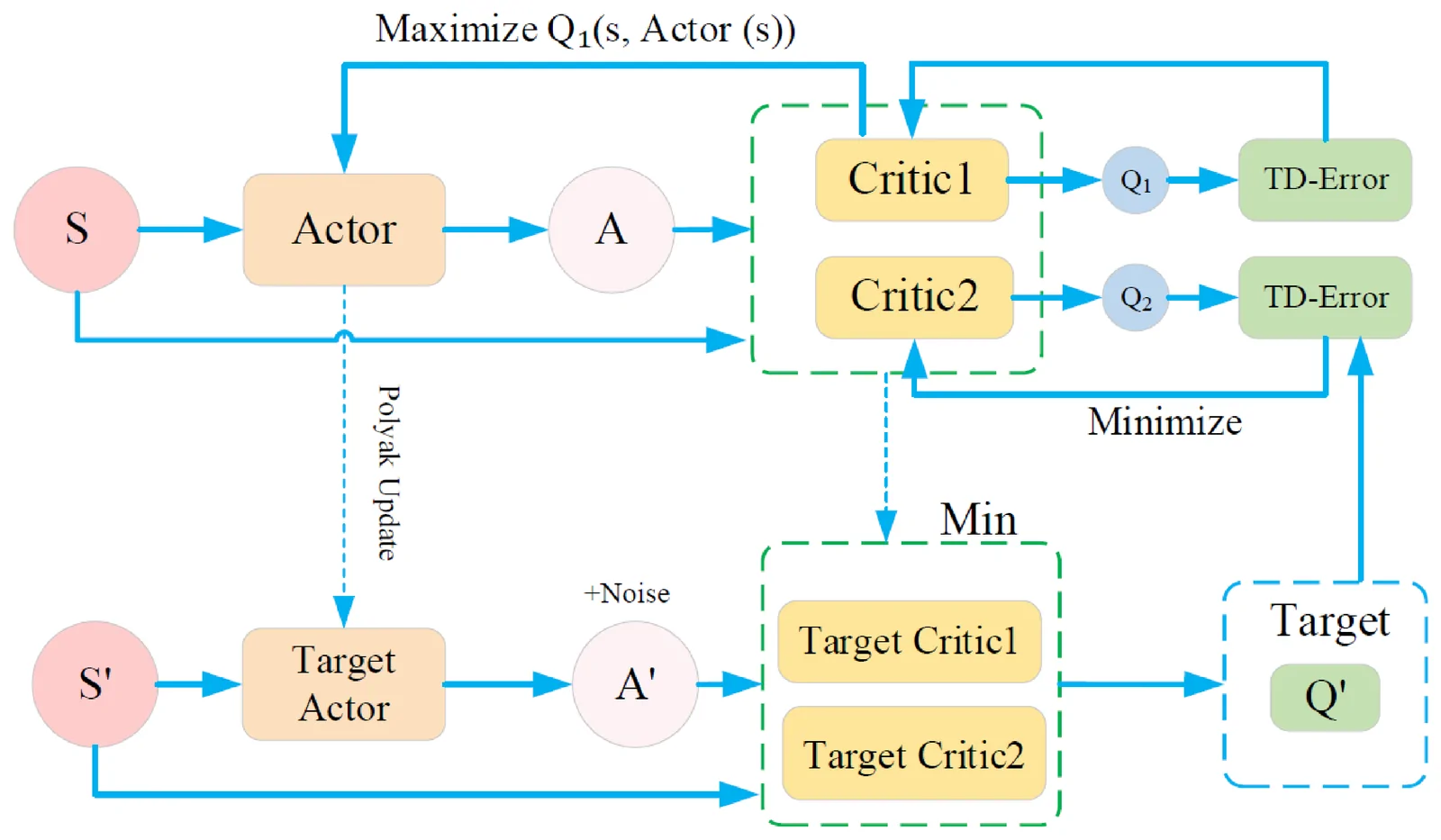
TD3 network architecture.

The TD3 algorithm [26] is a deep reinforcement learning method based on the Actor-Critic framework. It effectively mitigates the Q-value overestimation problem present in the DDPG algorithm [27], significantly improving algorithm stability and robustness. Its core design incorporates three key techniques: clipped double Q-learning, target policy smoothing, and delayed policy updates. The network structure is shown in Figure 6.

To suppress the overestimation bias of the Q-function, TD3 maintains two independent Critic networks $\left(Q_{\theta_{1}},Q_{\theta_{2}}\right)$ and their corresponding target networks $\left(Q_{\theta_{1}^{\prime }},Q_{\theta_{2}^{\prime }}\right)$. The target Q-value is generated as shown in Equation (12). The underestimated value is more difficult to propagate and amplify during backpropagation compared to the overestimated one, thereby providing a more conservative and stable target estimate:

y = *r_a_* + *γ_a_*min*Q_θ_*_′1_(*s*′,*a*′) (12)

where *r* is the reward signal induced by the action, *γ* is the discount factor, and (*s*′, *a*′) are the state and action at the next time step.

When TD3 computes the target Q-value, it adds a clipped normally distributed Gaussian noise to the target action to constrain the action perturbation range (|*ϵ*| ≤ *c*), thereby making the Q-function locally smooth in the action space and preventing the policy from overfitting to sharp peaks of the Q-function. By smoothing the dependence of the Q-value on actions, the policy network is forced to learn that a small region around the action yields a high Q-value, which increases the algorithm’s robustness and prevents overfitting to specific action peaks:

(13) $$a^{\prime }=\pi_{\phi^{\prime }}(s^{\prime })+\alpha ∼\mathrm{clip}(N)(0,\sigma ),(-c,c)$$

where *α* is random noise drawn from a normal distribution, clipped to the range (−*c*, *c*).

To enable harvesting path planning, both the Actor network and the Critic network constructed in this paper adopt a multilayer perceptron (MLP)-based architecture. In the 2D planning scenario, the agent’s own state is defined as *S_own_* ∈ ℝ^4^, $S_{\mathrm{own}}={\left[x,y,x_{g},y_{g}\right]}^{T}$, where (*x*, *y*) represents the current end-effector position and (*x_g_*, *y_g_*) denotes the target picking point position. In the 3D scenario, this is extended to $S_{\mathrm{own}}={\left[x,y,z,x_{g},y_{g},z_{g}\right]}^{T}$. The 2D obstacle state is defined as *S*_int_ ∈ ℝ^6^, $S_{\mathrm{int}}^{\left(i\right)}={\left[x_{i},y_{i},w_{i},h_{i},\Delta x_{i},\Delta y_{i}\right]}^{T}$, where (*x_i_*, *y_i_*) is the position of the i-th obstacle, (*w_i_*, *h_i_*) are its dimensions, and (Δ*x_i_*, Δ*y_i_*) is the offset relative to the agent. In the 3D scenario, $S_{\mathrm{int}}^{\left(i\right)}=\left[x_{i},y_{i},w_{i},h_{i},d_{i},\Delta ,x_{i},\Delta y_{i}\Delta z_{i}\right]$ additionally includes depth information *d_i_* for each obstacle. The Actor outputs a continuous action *a*_cont_ of dimension 2 and 3, respectively, which is discretized and mapped to the actual movement direction. In the 2D scenario, the azimuth angle of *a*_cont_ is computed and discretized into 8 primary directions plus a zero-direction (no movement). In the 3D scenario, based on the absolute magnitudes of the components of *a*_cont_, the action is discretized into positive and negative directions along the X-, Y-, and Z-axes, along with a stop action.

The hidden layer dimensions of all networks are uniformly set to 256. Layer normalization (LN) is applied for normalization, and residual connections are introduced to accelerate gradient propagation. The Critic network adopts the standard TD3 “twin-Q-network” structure, in which the two Q-networks share the same architecture but have independent parameters, serving to mitigate the Q-value overestimation problem.

#### 2.4.2. Attention Mechanism Optimization

**Figure 7:**
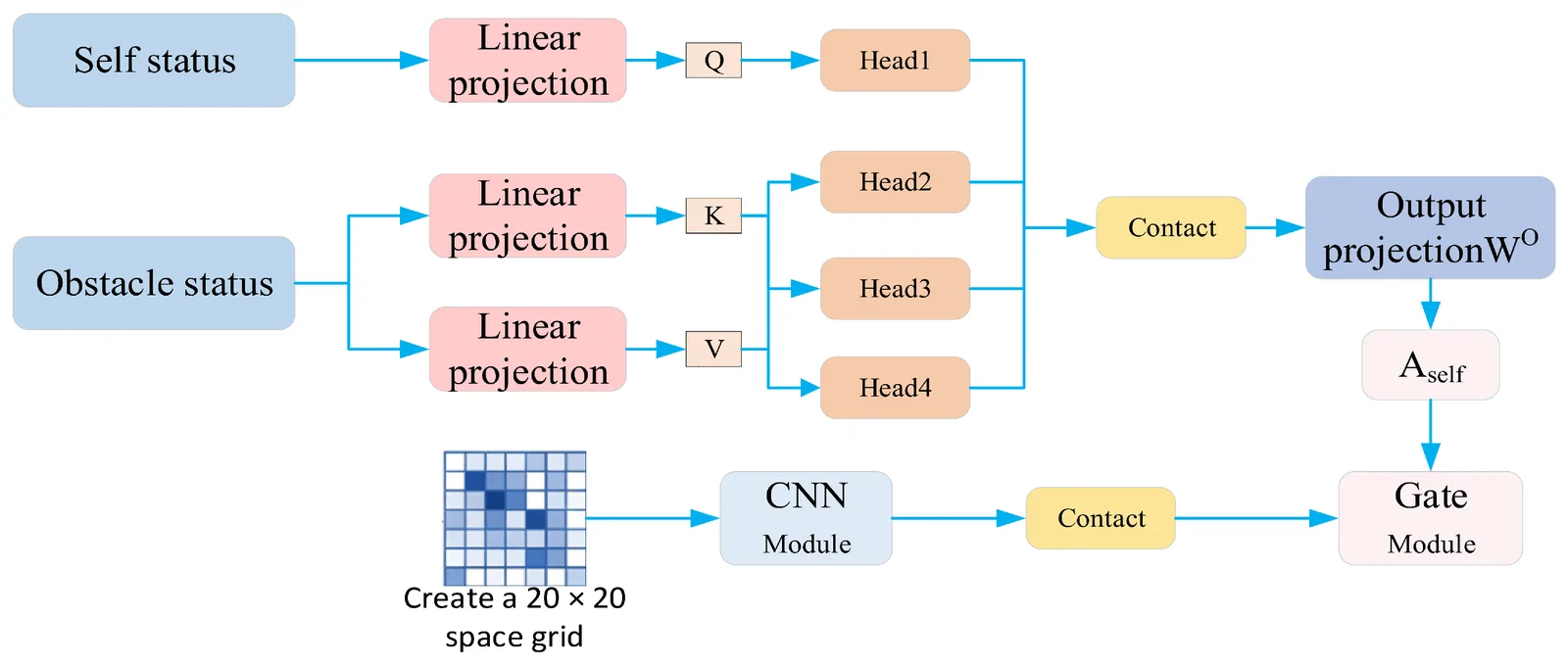
Structure of the multi-head attention mechanism.

A gated fusion mechanism is further incorporated into the attention mechanism introduced in this paper. The attention mechanism allows the algorithm to focus on the most relevant parts of the input; therefore, when fused with a gating mechanism, it enables more refined feature selection and fusion. This paper combines the multi-head attention mechanism [28] with a spatial gating fusion mechanism [29], allowing the algorithm to adaptively balance the two types of representations in each dimension. When obstacles are sparse, the attention branch is weighted more heavily; when obstacles are dense, the spatial grid branch is enhanced. In this way, global relational reasoning and local spatial perception are dynamically integrated, improving both the robustness of obstacle avoidance and path quality while avoiding decision errors. The structure of the multi-head attention mechanism is shown in Figure 7 below.

(1) Multi-Head Attention Mechanism

Let $X\in R^{T\times D_{\mathrm{in}}}$ and $Y\in R^{T^{\prime }\times D_{\mathrm{in}}}$ be two input matrices. Multi-head attention projects Q, K, and V into multiple subspaces, computes attention in parallel, then concatenates the results and obtains the final output via an output projection. Standard scaled dot-product attention is defined as shown in Equation (14); here, X is the agent’s own state projected as queries, and Y is the obstacle state matrix projected as keys and values:

(14) $$\mathrm{Attention}(Q,K,V)=\mathrm{softmax}(\frac{QK^{T}}{\sqrt{d_{k}}})V$$

where Q, K, and V are linear projections, the query matrix $W_{Q}\in R^{D_{\mathrm{in}}\times D_{k}}$, key matrix $W_{K}\in R^{D_{\mathrm{in}}\times D_{k}}$, and value matrix $W_{V}\in R^{D_{\mathrm{in}}\times D_{\mathrm{out}}}$ are parameterized. Attention applied to the same sequence (i.e., *X* = *Y*) is referred to as self-attention. Multi-head attention with *N_h_* heads is obtained by concatenating the attention results, as shown in Equations (15) and (16):

MultiHead(*X*, *Y*) = *Contact*(head*_i_*, …, head*_h_*)*W^O^* (15)

(16) $${\mathrm{head}}_{i}=\mathrm{Attention}(QW_{Q}^{\left(i\right)},KW_{K}^{\left(i\right)},VW_{V}^{\left(i\right)})$$

where *O* is the output sequence, head*_i_* is the *i*-th head, and each head *i* ∈ [*N_h_*] learns different parameter matrices ${W_{Q}}^{\left(i\right)},{W_{K}}^{\left(i\right)}\in R^{D_{\mathrm{in}}\times d_{k}},{W_{V}}^{\left(i\right)}\in R^{D_{\mathrm{in}}\times d_{\mathrm{out}}}$ along with an additional matrix $W_{O}\in R^{N_{h}d_{\mathrm{out}}\times D_{\mathrm{out}}}$, which projects the concatenated output of the *N_h_* heads into the output space $R^{D_{\mathrm{out}}}$. In the multi-head setting, *d_k_* denotes the dimension of each head, and *D_k_* = *N_h_d_k_* is the full dimension of the query space.

The first position of the output sequence, namely the attention enhancement feature of the corresponding agent itself, is taken as the global attention feature; the feature of the agent after global attention fusion is computed as shown in Equation (17):

(17) $$A_{\mathrm{self}}=\mathrm{MultiHead}(X,Y)[0,:]\in R^{d_{\mathrm{out}}}$$

In this paper, the number of multi-head attention heads is set to *N_h_* = 4, with the dimension of each head being *d_k_* = 64. As shown in Equations (14)–(16), the agent’s own state is projected to obtain the query matrix *Q*, while the obstacle states are projected to obtain the key matrix *K* and the value matrix *V*. The global correlation weights are then computed via scaled dot-product attention.

To compensate for the loss of fine local geometric relationships in attention-based feature extraction, a convolution-based spatial feature branch is introduced. A 20 × 20 grid is established in the environment, where each cell corresponds to a fixed-size spatial region. The agent’s position is always mapped to the grid center *c*. For a given obstacle *α*, its coordinates relative to the agent are mapped to (*g_x_*, *g_y_*), as given in Equations (18) and (19):

(18) $$g_{x}=c+\frac{\Delta x_{\alpha }}{S}$$

(19) $$g_{y}=c+\frac{\Delta y_{\alpha }}{S}$$

where *S* is the spatial scale factor, whose value should map the environmental extent to the grid range.

**Figure 8:**
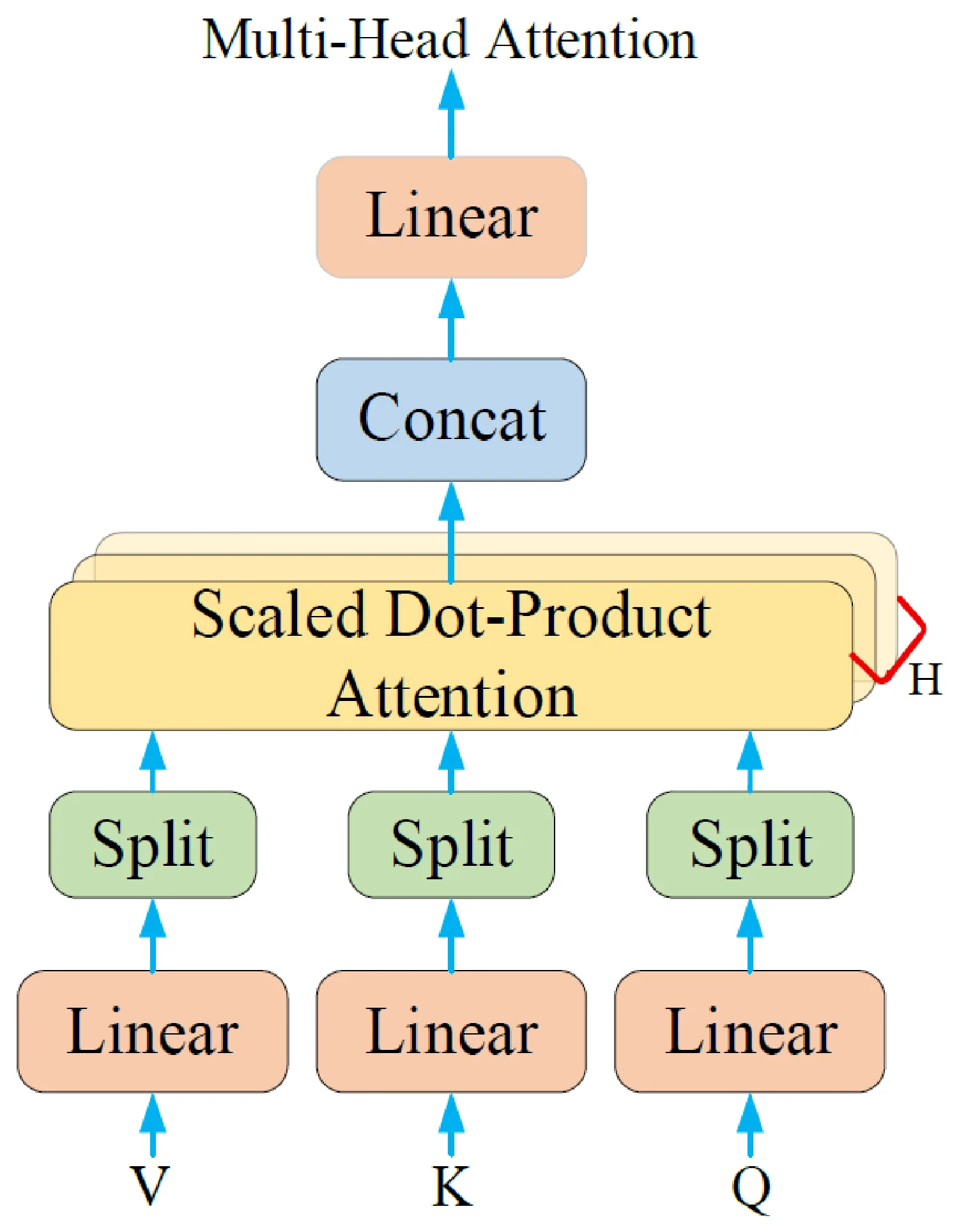
Multi-head attention mechanism.

Finally, a CNN feature extractor transforms the output feature map through a fully connected layer into a vector, ensuring that a fixed-size feature can still be output even if the grid size changes, thereby preserving model generalizability:

*f*_spatial_ = FC(CNN(grid_map); *W*_sp_, *b*_sp_) (20)

where *W*_sp_ is the fully connected weight of the spatial branch, *b*_sp_ is the fully connected bias of the spatial branch, and *f*_spatial_ is the final feature vector. The multi-head attention mechanism is illustrated in Figure 8.

In the spatial convolution branch, a 20 × 20 grid is constructed, with the agent fixed at the grid center, and obstacles are mapped to the grid according to Equations (18) and (19). This binary feature map is passed through two 3 × 3 convolutional layers, increasing the number of channels from 16 to 32. After adaptive average pooling, the feature map is flattened to a dimension of 32 × 8 × 8 = 2048, and then projected by a fully connected layer to the hidden dimension *d_h_* = 256, yielding the spatial feature *f*_spatial_.

(2) Gated Fusion Mechanism

In the gating mechanism, the agent’s own vector and the feature vector extracted by the CNN are concatenated into a joint feature vector. This vector is then passed through a fully connected layer followed by a sigmoid activation function to obtain the gating vector g. The agent’s own vector and the CNN-extracted feature vector are then combined element-wise linearly, as shown in Equations (21) and (22):

*g* = *σ*(*W_g_*[*A_self_*; *f_spatial_*] + *b_g_*) (21)

*f_out_* = *g* ⊙ *A_self_* + (1 − *g*) ⊙ *f_spatial_* (22)

where [;] denotes vector concatenation, *W*_g_ is the gating weight matrix, and *b*_g_ is the bias vector, *σ* is the sigmoid function, f_out_ is the final spatial attention feature, and ⊙ denotes element-wise multiplication. The resulting f_out_ has the same dimension as A_self_ and f_spatial_, i.e., 256.

**Figure 9:**
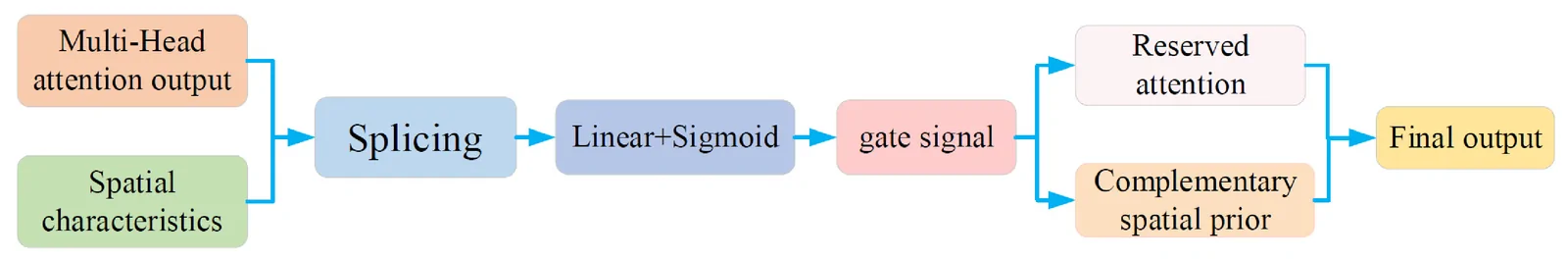
Schematic diagram of the gated fusion mechanism.

Each component *g_k_* of the gating vector determines the extent to which the output *f*_out_ along the *k*-th dimension is taken from *A*_self_, with the remainder taken from *f*_spatial_. When a component is close to 1, that dimension almost completely trusts the global attention; when it is close to 0, it relies on the local information extracted by the CNN. This dimension-wise soft gating gives the algorithm superior fusion capability and allows it to adapt to a variety of application scenarios. The schematic diagram is shown in Figure 9.

#### 2.4.3. Reward Function Optimization

In complex environments, deep reinforcement learning policies that rely solely on sparse task-oriented rewards may fail to focus on important features, leading to incorrect environmental feature extraction by the spatial perception module and consequently reducing robustness and generalization ability. Therefore, this paper introduces an attention-based spatial awareness reward term into the reward function. This reward term is used as a regularization signal to encourage the agent to maintain focus on critical information during learning. This reward enables the agent to more efficiently identify obstacles in a sparse reward space, thereby significantly improving path accuracy.

In this reward term, the attention probability distribution *P* of the agent over obstacles is first obtained from the attention module, and its information entropy is calculated as shown in Equation (23):

(23) $$H(P)=-\sum_{i=1}^{N}P_{i}\log(P_{i}+\delta )$$

The spatial awareness reward is then given by Equation (24):

*R*_spatial_ = −*λ_spatial_*|*H*(*P*) − *H_target_*| (24)

where *N* is the maximum number of obstacles, *δ* is a small smoothing constant, λ_spatial_ is the spatial perception weight, and p_i_ is the attention weight assigned to the i-th obstacle.

Here, λ_spatialis_ the spatial attention weight, and H_target_ is a constant target entropy. When the attention entropy approaches H_target_, the agent is able to focus on critical obstacles without completely ignoring other minor obstacles, thereby achieving a good trade-off in complex environments. At the same time, the reward function assigns a negative reward to any attention distribution that deviates from the target entropy—both overly concentrated and overly dispersed attention are penalized, thereby guiding the agent to learn to maintain a moderate level of attention.

The final total reward of the algorithm model is the weighted sum of the following components: goal-achievement reward, collision penalty, distance-change reward, path-length penalty, smoothness penalty, spatial attention reward, out-of-bounds penalty, and time-step penalty.

### 2.5. Expert Demonstration-Guided Safflower Filament Harvesting Path Optimization

Rewards in path planning are typically sparse. Therefore, this paper employs the improved RRT algorithm as a demonstration expert to provide dense prior data for the deep reinforcement learning algorithm. This significantly reduces the exploration space. Meanwhile, the twin Q-networks and target policy smoothing mechanism of TD3 effectively suppress overestimation and error accumulation when the reinforcement learning algorithm mimics expert demonstrations.

During path planning sampling, the proposed algorithm samples both expert samples and ordinary samples, using them only for the guidance loss. A critic expert guidance loss is added to the ordinary loss by maximizing the Q-value of expert actions. The calculation formulas are shown in Equations (25)–(27):

(25) $$L_{\mathrm{TD}}(\theta )=Ε\sim N\left[{\left(Q_{\theta }(s,a)-(r+\gamma \underset{i=1,2}{\min}Q_{\theta_{i}^{\prime }}(s^{\prime },\pi_{\phi^{\prime }}(s^{\prime })+\alpha )\right)}^{2}\right]$$

(26) $$L_{\mathrm{Expert}}(\theta )=-\frac{1}{B}\sum_{i=1}^{B}\underset{i=1,2}{\min}Q_{\theta i}(s^{\mathrm{ex}},a^{\mathrm{ex}})$$

*L_Critic_* = *L_TD_*(*θ*) + *w^e^L_Expert_*(*θ*) (27)

where *N* is the size of the ordinary samples; L_TD_ stands for temporal difference error loss; L_Expert_ denotes the loss for maximizing the Critic expert’s Q-value; *B* represents the expert batch size; s^ex^ and *a*^ex^ denote the expert’s demonstration state and action, respectively; and w^e^ denotes the expert guidance weight; r is the instantaneous reward value calculated and returned by the environment based on the reward function after executing action as in the current state s.

The total loss, L_Critic_(*θ*), includes the standard TD error term, L_TD_(*θ*), which forces the Critic to fit bounded target values generated by the target network. Since it shares the same network parameters as the expert loss, this creates a “regression constraint” on the Q-values to suppress their excessive inflation. Additionally, we employ gradient clipping, target policy smoothing, and a dual-Q mechanism to further limit the propagation of overestimates.

This loss makes the Critic believe that the expert yields higher returns, thereby guiding the Actor toward the expert in subsequent policy gradient updates. At the same time, the Actor is forced to imitate the expert’s actions, maintaining output actions consistent with the expert in regions visited by the expert. This constrains the output of the policy network and prevents it from deviating significantly from the expert region during the early stages of training.

To enable the Actor to effectively imitate expert demonstrations, we introduce a behavioral cloning loss into the Actor’s objective function. The overall Actor loss is defined as:

(28) $$L_{\mathrm{Actor}}=-\frac{1}{N}\sum_{i=1}^{N}Q_{\theta 1}(s_{i},\mu (s_{i}))+w^{e}\cdot \frac{1}{B}\sum_{j=1}^{B}{\left\|\mu (s_{j}^{\mathrm{ex}})-a_{j}^{\mathrm{ex}}\right\|}^{2}$$

where $Q_{\theta_{1}}$ is the value estimate of the first Critic network for the state-action pair; and *μ*(*s_i_*) is the continuous action output by the Actor network given the current state.

## 3. Results and Discussion

### 3.1. Environment Configuration

To verify the effectiveness of the proposed path planning algorithm for the safflower filament harvesting robot, all algorithms in this paper were tested under the following environment configuration. The operating environment is Windows 11 (64-bit), with a computer configuration of an AMD Ryzen Threadripper PRO 3975WX 32-core processor at 3.50 GHz, 384 GB RAM, and an NVIDIA RTX A5000 GPU with 24 GB VRAM. The deep learning framework used is PyTorch 1.13, the development environment is Python 3.8, and the computing framework is CUDA 11.7. All compared algorithms were trained with identical random seeds and the same number of training episodes to ensure a fair comparison.

### 3.2. Algorithm Training

The improved algorithm in this paper first uses the improved RRT planner to generate expert paths for each start–goal pair. After Douglas-Peucker simplification and Shortcut optimization, the state–action pairs on the paths are stored in the expert demonstration buffer. During training, the robot explores in the environment episode by episode. At each step, its own state and obstacle information are fed into the spatial attention Actor network, which outputs continuous actions. These actions are discretized and executed as movements, and an immediate reward is calculated according to the reward function. The data from each step are stored in the experience replay buffer. When the buffer has sufficient samples, ordinary samples and expert samples are mixed in a predetermined ratio to form training batches. The Critic network and Actor network are updated sequentially, and the target networks are softly updated. The training parameters are listed in Table 2.

The learning rate and discount factor adopt the default recommended values from the TD3 literature, and their effectiveness was verified through small-scale grid search in the safflower harvesting scenario. The target network soft update coefficient and the policy delay update steps follow the standard TD3 configuration to ensure training stability. The batch size and replay buffer capacity were determined by jointly considering GPU memory constraints and the demand for sample diversity. The expert sample ratio and guidance weight were determined based on ablation experiments, achieving a favorable balance between “guidance strength” and “policy exploration freedom.” The spatial attention reward weight was set to a relatively small value, serving as an auxiliary regularization signal to guide the attention distribution rather than dominating decision-making.

**Figure 10:**
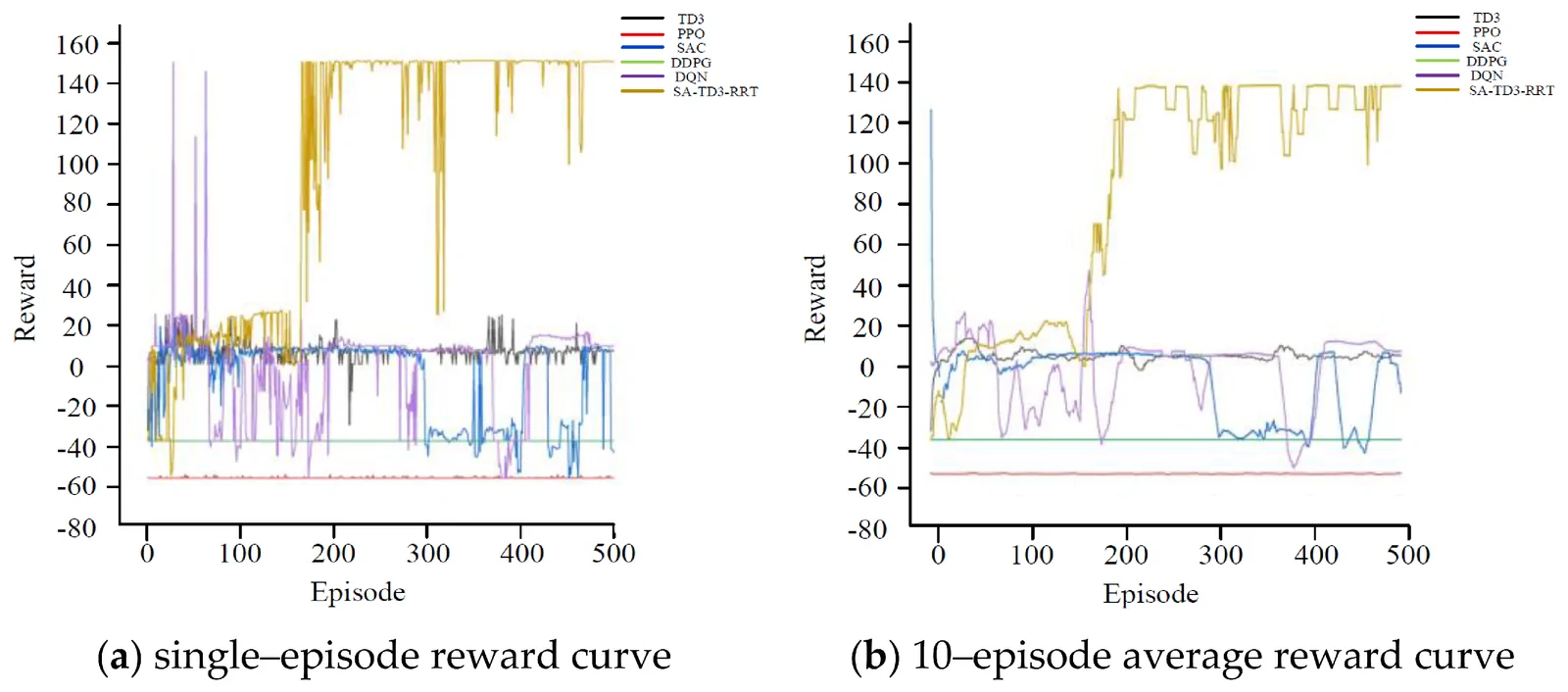
Reward curves.

During training, the same training settings were used to train the TD3 variants (before and after improvement) as well as common deep reinforcement learning algorithms. However, expert demonstration is not used in the baseline algorithm. The reward curves for each algorithm are shown in Figure 10.

Under the same training settings, the proposed SA-TD3-RRT algorithm was compared with mainstream deep reinforcement learning algorithms such as TD3, PPO, SAC, DDPG, and DQN for the safflower filament harvesting robot path planning task. SA-TD3-RRT exhibits a rapid reward increase in the early training stage (approximately the first 100 episodes), with convergence speed significantly outperforming the other algorithms. Its 10-episode moving average reward curve shows relatively small fluctuations, indicating good training stability. In contrast, although TD3 and SAC converge relatively quickly, they still exhibit noticeable jitter in the mid-to-late stages; PPO converges at a moderate speed, while DDPG and DQN converge slowly with substantial fluctuations. In terms of the final reward value, SA-TD3-RRT reaches approximately 160 by the end of 500 training episodes, which is significantly higher than TD3 (≈140), SAC (≈120), PPO (≈110), and DDPG and DQN (both below 100). These results fully validate the advantages of the SA-TD3-RRT algorithm in combining RRT-guided exploration with TD3 policy optimization in sparse reward environments, effectively improving both convergence performance and policy stability for path planning tasks.

### 3.3. Algorithm Planning Experiments

To evaluate the overall path planning performance of the proposed algorithm, two-dimensional and three-dimensional path planning was conducted in a simulation environment for the task of picking up safflower fibers, and the comprehensive performance of the proposed SA-TD3-RRT algorithm in picking path planning was systematically evaluated. To ensure the robustness of the statistical results, a trimmed mean method is adopted in this paper for the statistical analysis of planning time, path length, and number of path turning points across multiple experimental trials. For a given set of experimental data, the values are first sorted in ascending order, after which the maximum and minimum values are removed. The mean and standard deviation are then computed from the remaining data, which effectively reduces the influence of outliers on the overall statistical results. To ensure that the compared algorithms are evaluated under identical initial conditions, the random seed is fixed throughout this study.

It is important to note that all raw paths generated by different algorithms were subjected to an identical post-processing pipeline, consisting of Douglas-Peucker simplification, Shortcut optimization, and wandering segment removal. This ensures that the reported reductions in path nodes reflect genuine improvements in path topology and efficiency, rather than artifacts of algorithm-specific preprocessing.

#### 3.3.1. Ablation Experiments

To verify the effectiveness of the proposed improvements, components were added sequentially to the TD3 algorithm. The results are shown in Table 3. In the table, 3D post-processing node refers to the number of nodes remaining after applying the same post-processing pipeline (Douglas–Peucker simplification, shortcut deletion, and circular detection) to each original path generated by the algorithm. This value reflects the total number of path points after post-processing, rather than the actual number of path turning points output by the algorithm.

It should be noted that the ablation experiment in this section is used to separate the incremental contributions of various components within the proposed framework; The comparative experiment in Section 3.3.2 evaluates the complete SA-TD3-RRT framework. Since RRT-based expert guidance is an integral part of the framework, it is not added to the baseline reinforcement learning algorithm. Therefore, the performance difference in the comparative experiment reflects the joint effect of improved RRT expert guidance, spatial attention, and gating fusion, which should not be attributed only to the network architecture.

As shown in the table, in 2D planning, compared to the baseline TD3 algorithm, adding the attention mechanism reduces the number of path nodes by 85.97% and shortens the path length by 69.46%, while planning time increases only slightly by 13.3%. The significant reduction in path complexity greatly facilitates subsequent path smoothing and real-time execution, while the additional computational overhead remains within an acceptable range for offline planning or precomputation scenarios. In 3D planning, the planning time was reduced by 48.33%, and the path length also decreased slightly. This indicates that the attention mechanism enables the algorithm to focus more on task-relevant features, greatly improving path planning performance.

Subsequently, the gated fusion mechanism was further added on top of the attention mechanism. The experiments show that, based on the attention mechanism, this mechanism shortens the 2D path length by 4.86%. Although the planning time increased slightly, the number of turning points was reduced from 1130 to 13. In 3D planning, the planning time was reduced while the path length was also decreased by 16.15%. The adaptive information fusion of this mechanism leads to smoother paths.

Finally, the improved RRT is incorporated into the algorithm as an expert demonstration, further reducing both planning time and path length. In 2D planning, the time is only 3.65 s, the path length is reduced by 85.64% compared to the TD3 algorithm, and the number of path turning points remains at 13. In 3D planning, the planning time is shortened from 25.16 s to 4.72 s, and the path length is reduced by 2.29%. The improved algorithm significantly reduces path length while maintaining planning efficiency. In both 2D and 3D planning tasks, the path length is decreased. Although the path length reduction in 3D planning is 2.29%, the planning time is reduced by 81.24%, greatly enhancing real-time performance.

#### 3.3.2. Comparative Experiments with Other Deep Reinforcement Learning Algorithms

**Figure 11:**
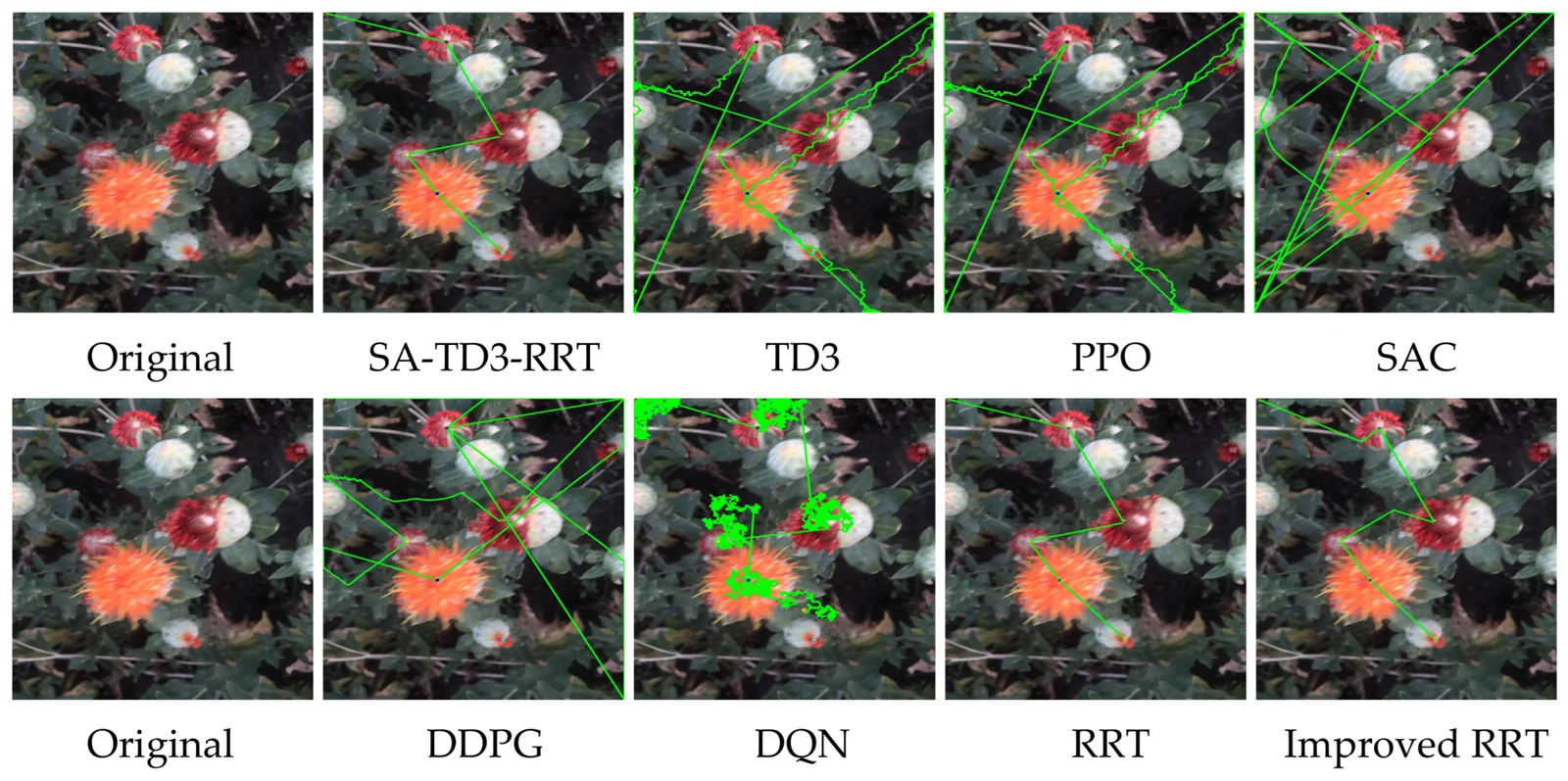
The 2D planning results of different deep reinforcement learning algorithms.

**Figure 12:**
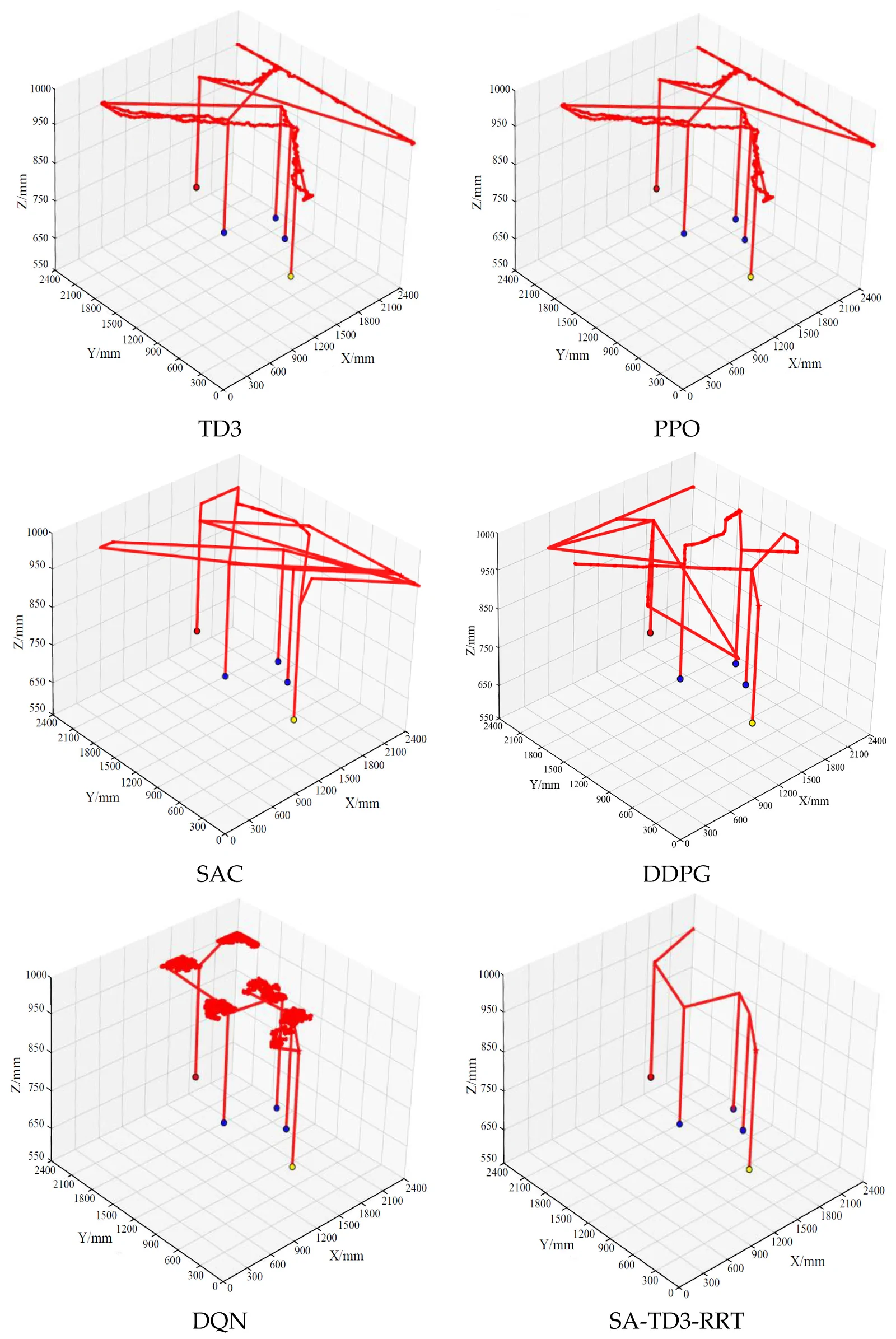

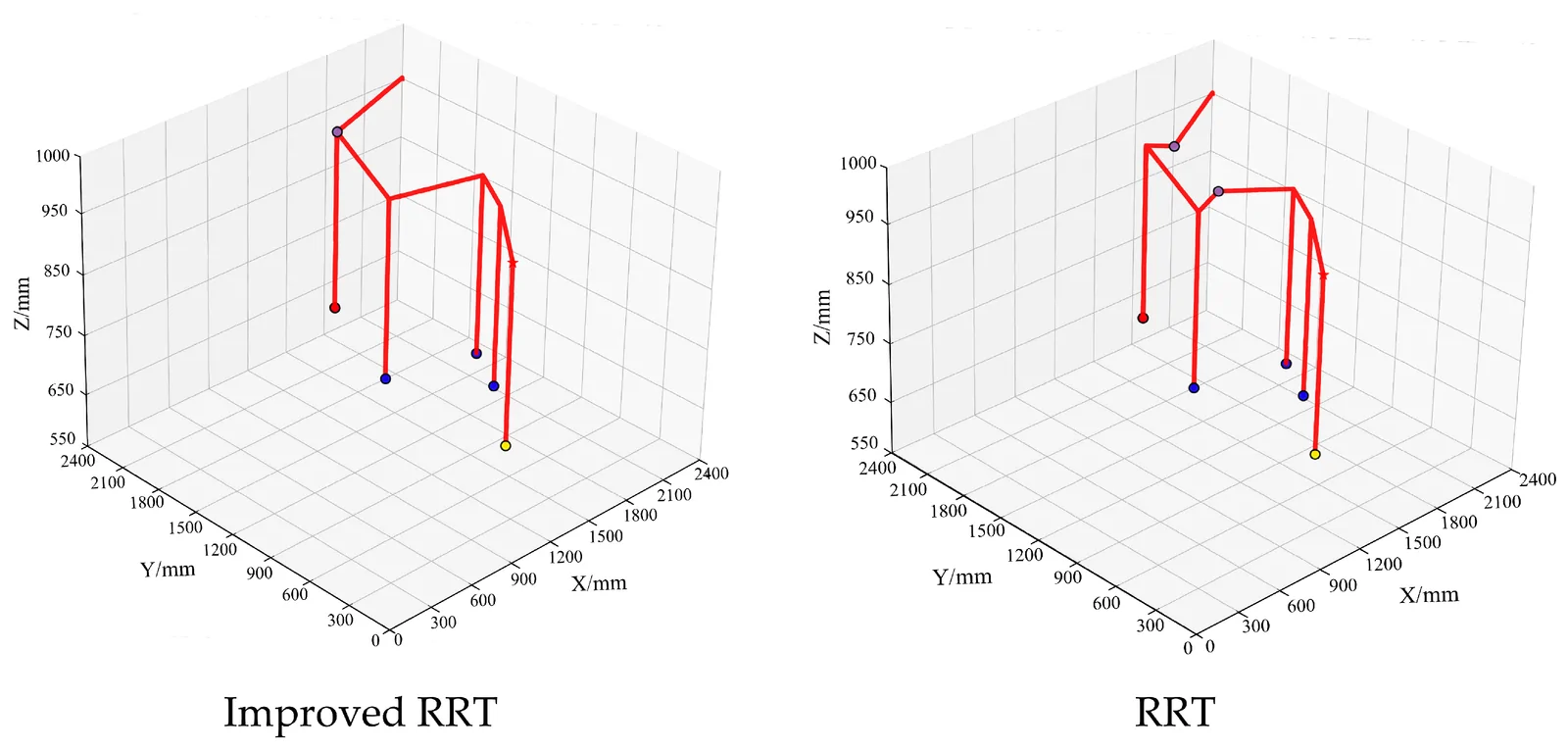
The 3D planning results of different deep reinforcement learning algorithms.

To verify the superiority of the proposed algorithm, it was compared with deep reinforcement learning algorithms such as TD3 [26], PPO [30], SAC [31], DDPG [27], and DQN [32], as well as traditional algorithms such as RRT and RRT enhanced by the method described in this paper. The experiments were conducted in multiple safflower harvesting path planning environments. Each algorithm underwent 100 planning trials in the same environment, and the average results were taken as the final outcomes. The results are shown in Table 4. The two-dimensional planning results are shown in Figure 11, and the three-dimensional planning results are shown in Figure 12.

All baseline reinforcement learning algorithms were trained in their standard configuration without using the expert demonstration generated by RRT. Therefore, this comparison measures the overall benefit of the complete SA-TD3-RRT process under the same training and round budget, rather than claiming that the architecture modification alone led to the observed gain. The ablation results in Table 3 provide some evidence for the individual incremental contributions of attention, gating, and expert guidance within the proposed framework.

The experimental data and the planned paths shown in the figures above indicate that the improved algorithm performs well in both 2D and 3D planning tasks. In the 2D environment, the path length is shortened from 3687.47 mm (TD3) to 529.52 mm, which is only 14.36% of that of TD3, while the planning time is also reduced by 17.68%, reaching 3.65 s. Compared with other reinforcement learning algorithms, the path length reduction exceeds 80%, being only 7.45% of PPO, 15.94% of SAC, and 17.31% of DDPG. Along with the reduction in path length, path quality is also improved: the number of turning points decreases from 8053.9 to 13. Furthermore, in 3D planning, the improved algorithm also shows a significant improvement. The planned path length is shortened from 20,195.78 mm (TD3) to 18,520.92 mm, a reduction of 8.29%. Although the path planning time is slightly higher than that of SAC, it still reaches 4.72 s, outperforming DDPG, DQN, and other algorithms. Compared with the baseline RRT algorithm, the proposed algorithm achieves shorter paths in both 2D and 3D environments. Meanwhile, the improved RRT algorithm alone reduces planning time, path length, and the number of path nodes relative to the baseline RRT. After TD3 learning, the path length is further reduced, which to some extent demonstrates that the TD3 learning module enhances the path planning speed.

#### 3.3.3. Statistical Significance Validation

To further verify whether the differences in performance metrics among the aforementioned algorithms are statistically significant, this study conducted a Kruskal–Wallis H test on the path length and planning time data for all compared algorithms in a two-dimensional environment.

Table 5 shows that, in a two-dimensional scenario, there are highly significant differences among the various algorithms in terms of path length and planning time. Subsequently, post hoc pairwise comparisons were conducted using the Dunn test, and Bonferroni correction was applied to control the family-wise error rate for multiple comparisons. The results show that the path length of the SA-TD3-RRT algorithm is significantly shorter than that of TD3, PPO, SAC, DDPG, and DQN, and its planning time is also significantly shorter than that of all comparison algorithms except PPO and the improved RRT. These results statistically confirm that the proposed hierarchical framework achieves genuine and reliable improvements in path planning performance.

### 3.4. Path Planning Comparison in Different Environments

**Figure 13:**
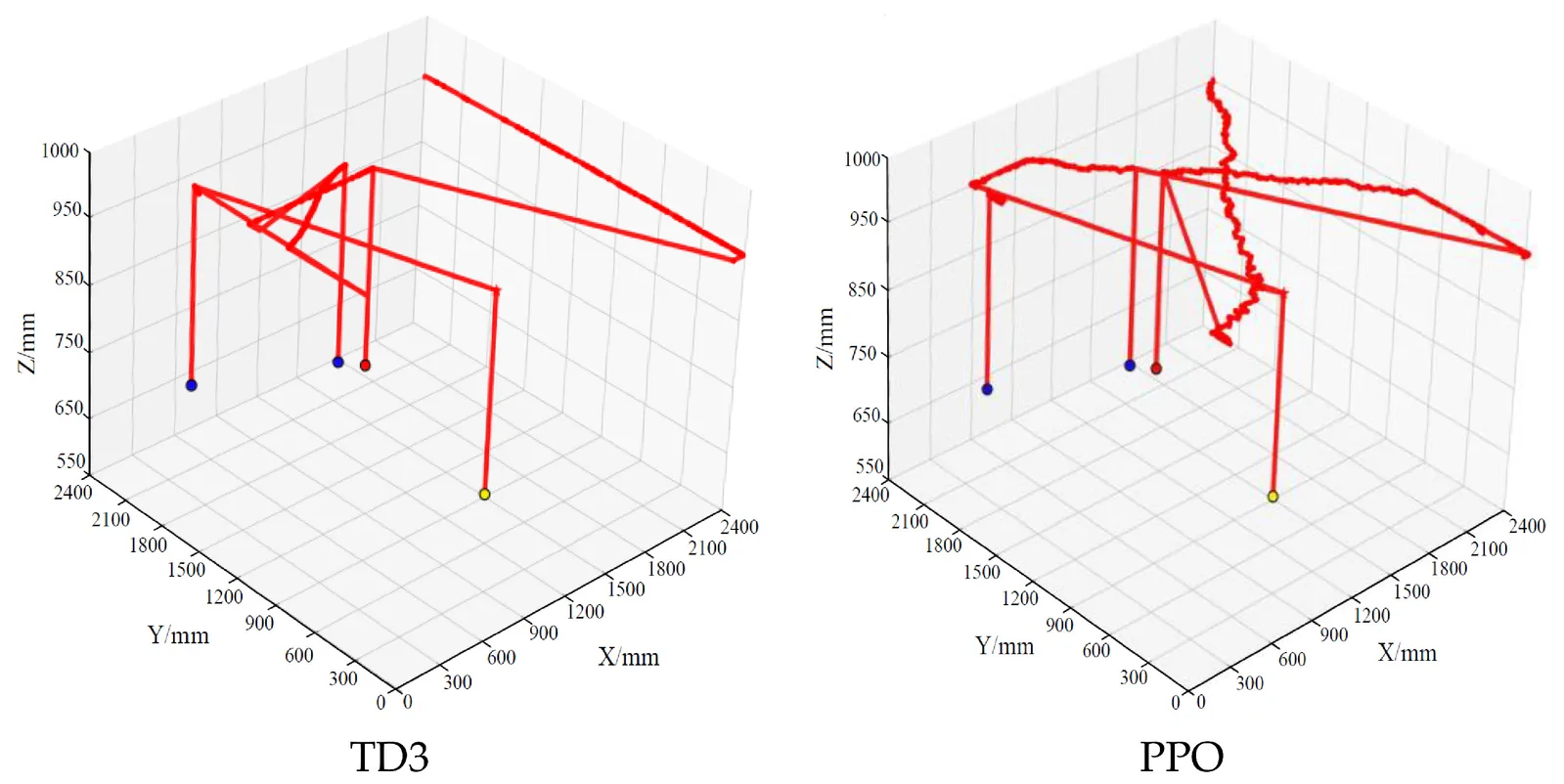

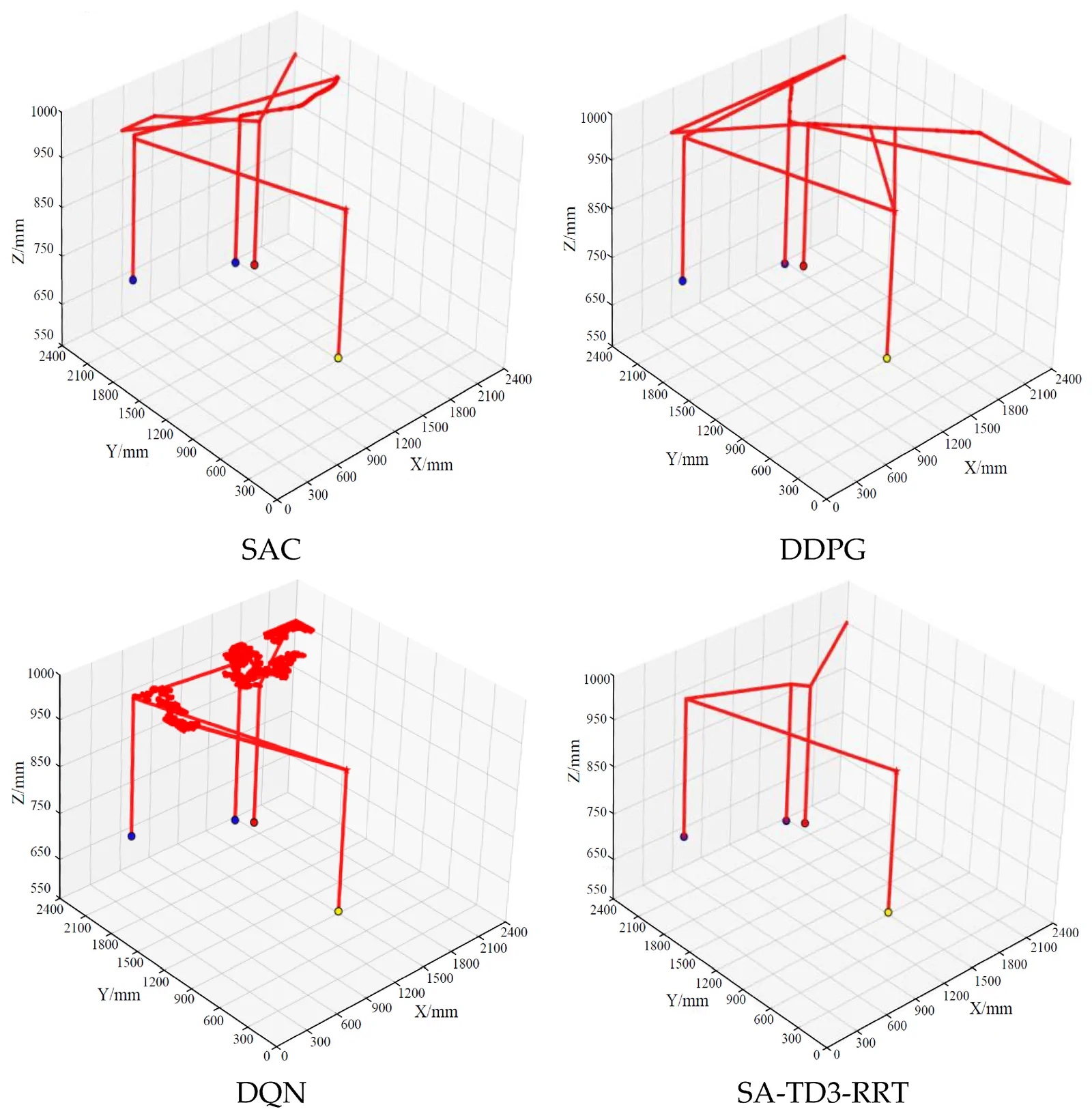
The 3D planning results in a sparse-target environment.

**Figure 14:**
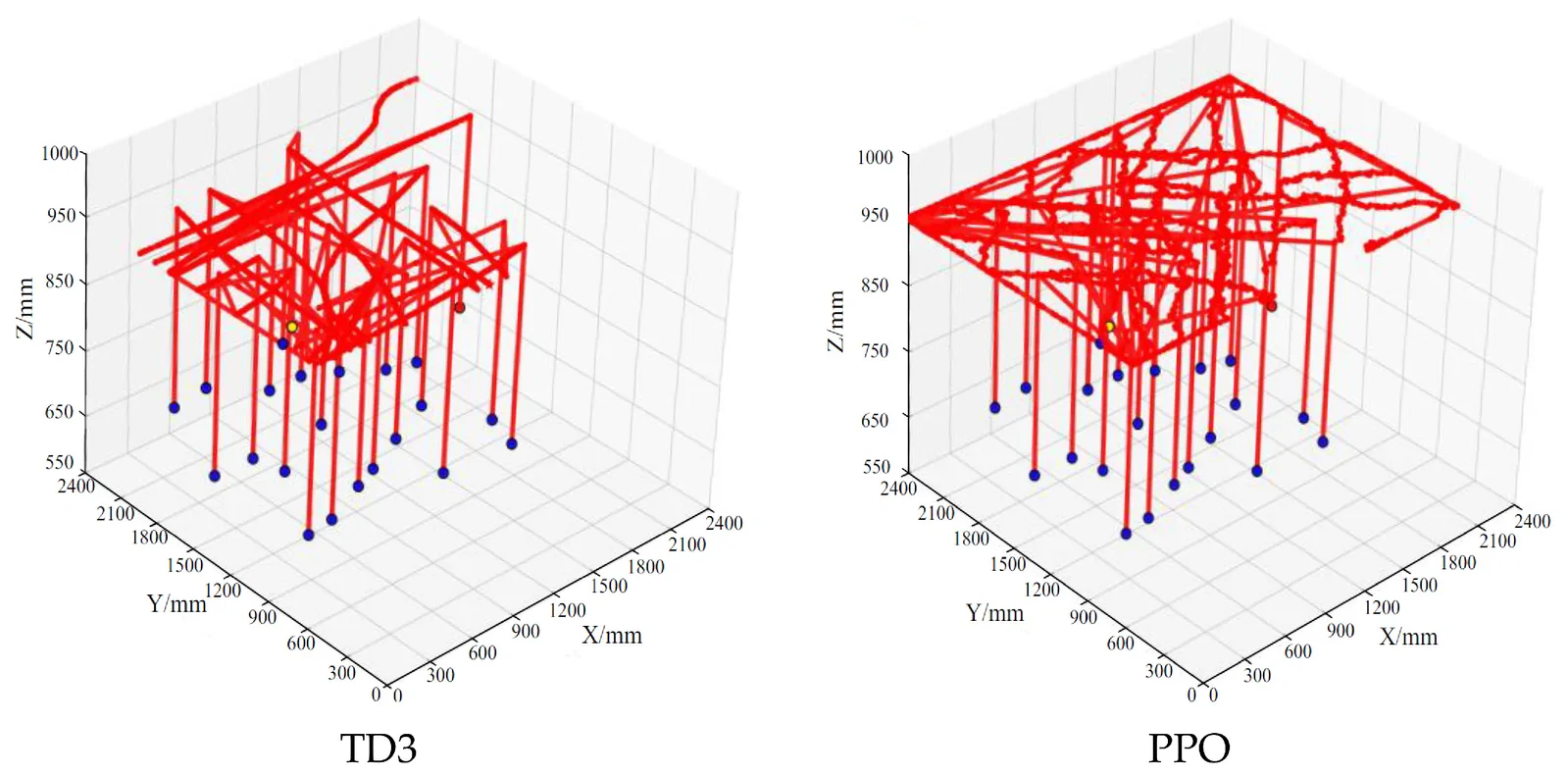

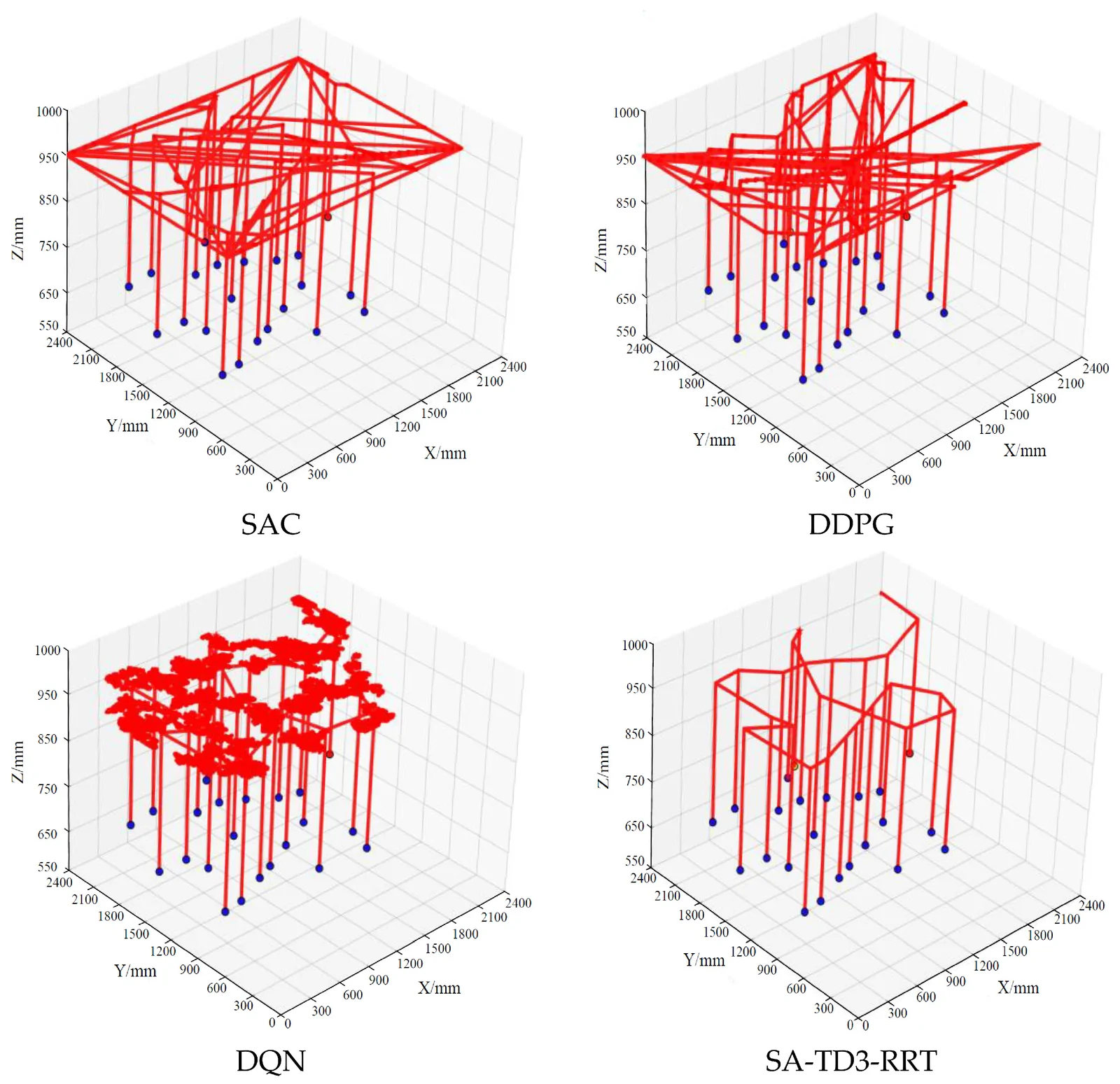
The 3D planning results in a multi-target environment.

To comprehensively evaluate the overall performance of the proposed SA-TD3-RRT algorithm for safflower filament harvesting path planning in complex environments, tests were conducted in two typical scenarios: simple environments and complex environments. The algorithm was compared with several mainstream path planning algorithms. The results are shown in Table 6 and Table 7. The 3D planning results are illustrated in Figure 13 and Figure 14.

Among them, the sparse-target environment contains 3–5 safflower filament harvesting target points with an average spacing between target points greater than 100 mm, and 2–3 obstacles; the multi-target environment contains 8–12 harvesting target points distributed in clusters with an average inter-target spacing of less than 50 mm, and 4–6 obstacles.

The data in Table 6 and Table 7 show that for 2D path planning in a simple safflower growth environment, the proposed algorithm demonstrates excellent performance in path length optimization. The path length is only 37.17% of that of TD3 and is reduced by 85.15%, 62.26%, 60.67%, and 90.57% compared to PPO, SAC, DDPG, and DQN, respectively. Although the planning time is slightly longer than that of SAC and DDPG, it remains at a high performance level of only 2.94 s, while the number of turning points is dramatically reduced to only 8. In 3D planning, the planning time is significantly reduced from 18.96 s (TD3) to 3.13 s. Although the number of 3D post-processed nodes does not decrease, the path length is shortened by 25.99%, 8.22%, 8.30%, and 36.65% compared to PPO, SAC, DDPG, and DQN, respectively. In summary, the SA-TD3-RRT algorithm shows clear advantages in simple environments, especially in 2D path planning where the path length improvement exceeds 60%.

In the complex safflower growth environment, compared with TD3, the 2D path length is reduced by 62.83%, and compared with PPO, SAC, DDPG, and DQN, the improvement exceeds 85%. The planning time is reduced from 8 s to 1.74 s, and the number of path nodes is only 46. In 3D planning, the planning time is reduced by 77.37% to 17.62 s, and the path length is shortened by 30.89%, 15.04%, 15.32%, and 39.95% compared to PPO, SAC, DDPG, and DQN, respectively. Although the planning time is slightly higher than that of SAC and DDPG in both 2D and 3D planning, the algorithm still maintains good path planning quality.

The results shown in Figure 13 and Figure 14 indicate that in the sparse-target environment for safflower filaments, the improved SA-TD3-RRT algorithm significantly reduces the 3D path length, whereas algorithms such as PPO, SAC, DDPG, and DQN exhibit issues such as deviation from the target point and path repetition during path planning. In the complex multi-target environment, compared with PPO, SAC, DDPG, and DQN, the SA-TD3-RRT algorithm substantially reduces the degree of path repetition, achieves more accurate targeting toward the goal point, greatly reduces path complexity, and better meets the performance requirements of a safflower filament harvesting robot in complex natural environments.

In summary, the SA-TD3-RRT algorithm maintains clear advantages in path length optimization in both simple and complex environments. Although the path planning time increases slightly, the increase is not substantial. The data indicate that the stability of the SA-TD3-RRT algorithm has obvious advantages, and it still maintains good performance in complex environments. The improved algorithm significantly enhances path quality while balancing planning efficiency.

## 4. Conclusions

To address the challenges of safflower harvesting in complex, unstructured growth environments—where traditional path planning algorithms suffer from poor real-time performance and a tendency to fall into local optima, while pure deep reinforcement learning algorithms exhibit low training efficiency, sparse rewards, insufficient spatial perception, and difficulty balancing real-time planning and quality—this paper proposes a hierarchical path planning algorithm named SA-TD3-RRT. This algorithm integrates an improved RRT with a spatial attention-enhanced TD3, enabling efficient, low-loss path planning for safflower harvesting robots. Through theoretical analysis and simulation experiments, the feasibility and effectiveness of the proposed algorithm were rigorously validated within the simulation framework. The main conclusions are as follows:

(1) The traditional RRT algorithm was improved in multiple aspects: a goal-biased dynamic decay strategy was introduced to guide the search tree toward the goal while reducing redundant nodes; an adaptive step-size mechanism was designed to achieve “fast exploration in the early stage and fine convergence in the later stage,” thereby improving search efficiency; and a three-stage path post-processing pipeline combining Douglas-Peucker simplification, Shortcut straightening, and wandering detection was implemented to achieve near-optimal path length while ensuring path feasibility. The high-quality paths generated can serve as expert demonstrations, providing prior experience for reinforcement learning.
(2) A spatial attention-gated fusion module was constructed on top of the TD3 algorithm. A multi-head self-attention mechanism captures global obstacle distribution relationships, a convolutional spatial branch extracts local geometric features, and dimension-wise soft gating achieves adaptive fusion of the two feature types. Furthermore, a spatial awareness term was added to the reward function to guide the model to focus on key environmental features, further improving obstacle-avoidance capability and path smoothness.
(3) An expert guidance training mechanism based on imitation learning was proposed. Smooth paths generated by the improved RRT are converted into state-action pairs and stored in an experience replay buffer. By mixing ordinary samples with expert samples and designing a Critic expert guidance loss, the issues of blind exploration and slow convergence in the early stages of reinforcement learning training are effectively resolved, significantly reducing the policy’s exploration space.
(4) Simulation results show that, in the simulation environment, the proposed SA-TD3-RRT algorithm achieves significant performance advantages in safflower harvesting scenarios of varying dimensions and complexities. In 2D environments, the planned path length is reduced by 85.64%, 82.69%, and 92.55% compared to TD3, DDPG, and PPO, respectively; planning time is shortened by 17.68% to 3.65 s; and the number of path nodes is compressed from thousands to 13. In 3D environments, the path length is reduced by 8.29% compared to TD3, and planning time is drastically reduced by 81.24% to 4.72 s, meeting real-time operational requirements. In comparative tests between sparse-target simple environments and multi-target complex environments, the algorithm consistently maintains a significant advantage in path length, and its performance degradation in complex environments is much smaller than that of other comparison algorithms, demonstrating good stability and generalization capability.

In summary, the proposed algorithm significantly improves search real-time performance, although there is still room for enhancement in path smoothness and multi-target real-time performance. Future research will further introduce path optimization mechanisms and explore 3D path optimization strategies to improve both real-time performance and path quality in three-dimensional planning. Future work will explore the construction of a hybrid reinforcement learning architecture that integrates the continuous policy gradient model (TD3) proposed in this paper with a discrete heuristic switching layer. Specifically, the discrete switching layer outputs high-level decision commands based on the environmental state, selecting the next target to be harvested or switching the motion mode; the continuous policy network then generates specific motion actions under the constraints of these commands. The two layers are jointly trained through a hierarchical reinforcement learning framework, with the discrete layer responsible for global task allocation and path topology optimization, and the continuous layer responsible for local trajectory tracking and refined obstacle avoidance, thereby improving both the real-time performance and adaptability of task planning in multi-target environments.

We will systematically integrate physical factors—such as kinematics, hardware dynamics, and noise in the vision processing pipeline—by embedding the planner into the complete robot control stack, incorporating real-time depth-based uncertainty, and adjusting safety constraints to align with realistic tolerance ranges. We will conduct field experiments on real safflower plants to validate the algorithm’s effectiveness under actual agricultural conditions. At the same time, we will also combine the RRT-based expert guidance mechanism with other reinforcement learning baselines to systematically evaluate its versatility and further improve the multi-objective real-time performance.

## Figures and Tables

**Table 1:** Variable and function specifications.

Definition	Description
S_own_	Agent’s own state
S_int_	Obstacle states
mask	Valid obstacle mask
a_cont_	Continuous action output by the Actor
a_disc_	Discretized execution action
CollisionFree (node)	Collision detection function
Reward (…)	Reward function
Backtrack (n, P)	Backtracking mechanism

**Table 2:** Training parameters.

Parameter	Value
Spatial grid size	20 × 20
Learning rate	3 × 10^−4^
Discount factor	0.99
Target network soft update coefficient	0.005
Policy delay update steps	3
Batch size	640
Expert sample ratio	0.2
Expert guidance weight	0.3
Spatial attention reward weight	0.1
Maximum steps per episode	500
Number of training episodes	500
Adam	all networks
Gradient-Clipped Threshold Crop Threshold	1.0
Target Action Noise	0.2
Noise Clipping	0.5
Experience Pool Capacity	20,000
Goal-achievement reward weight	10
Collision penalty weight	10
Distance-change reward weight	0.5
Path-length penalty weight	0.1
Smoothness penalty weight	0.02
Spatial attention reward weight	0.1
Out-of-bounds penalty weight	0.01
Time-step penalty weight	0.01
Behavioral cloning weight	0.3

**Table 3:** Ablation experiment results.

Model	Attention	Gating	Expert Guidance	2D Path Average Search Time (s)	2D Path Average Length (mm)	2D Path Nodes	3D Path Average Search Time (s)	3D Path Average Length (mm)	3D Post-Processed Nodes
TD3	×	×	×	4.43 ± 0.12	3687.47 ± 227.03	8053.9 ± 16.2	25.16 ± 0.02	20,195.78 ± 227.03	41,293
	√	×	×	5.02 ± 0.03	1126.14 ± 69.45	1130 ± 136.9	13 ± 2.42	22,739.59 ± 465.71	41,293
	√	√	×	5.86 ± 1.99	1076.29 ± 46.40	13 ± 1.4	7.28 ± 2.66	19,067.69 ± 167.14	40,578
	√	√	√	3.65 ± 0.19	529.52 ± 6.40	13	4.72 ± 0.11	18,520.92 ± 120.20	40,578

**Table 4:** Comparative experimental results of different deep reinforcement learning algorithms.

Algorithm	2D Path Average Search Time (s)	2D Path Average Length (mm)	2D Path Nodes	3D Path Average Search Time (s)	3D Path Average Length (mm)	3D Post-Processed Nodes
TD3	4.43 ± 0.12	3687.47 ± 227.03	8053.9 ± 16.2	25.16 ± 0.02	20,195.78 ± 291.82	41,293
PPO	1.33 ± 0.09	7107.65 ± 80.9	5897.3 ± 165.43	13.9 ± 0.03	24,499.97 ± 432.60	41,293
SAC	4.3 ± 0.20	3310.78 ± 158.94	5196.6 ± 130.42	3.0 ± 0.06	21,302.18 ± 278.62	41,293
DDPG	4.8 ± 0.07	3059.04 ± 61.11	5551.1 ± 213.54	5.0 ± 0.06	21,507.78 ± 420.14	41,293
DQN	5.0 ± 0.04	11,166.45 ± 293.61	5055.2 ± 94.79	8.0 ± 0.04	28,298.07 ± 371.25	41,293
RRT	4.6 ± 0.02	538.36 ± 6.68	13.1 ± 0.7	20.96 ± 0.04	18,529.93 ± 100.61	37,021
Improved RRT	2.4 ± 0.01	535.47 ± 4.55	15.9 ± 0.3	2.3 ± 0.03	18,526.40 ± 155.62	36,783
SA-TD3-RRT	3.65 ± 0.19	529.52 ± 6.40	13	4.72 ± 0.11	18,520.92 ± 120.20	40,578

**Table 5:** Adjusted *p*-values and effect sizes for pairwise comparisons in 2D planning.

Comparison	Dimension	Adj. *p*-Value	Effect Size (r)
SA-TD3-RRT vs. TD3	2D Path search time	<0.001	0.97
	2D Path length	<0.001	0.983
SA-TD3-RRT vs. PPO	2D Path search time	<0.001	≈0.99
	2D Path length	<0.001	≈0.99
SA-TD3-RRT vs. SAC	2D Path search time	<0.001	≈0.99
	2D Path length	<0.001	0.942
SA-TD3-RRT vs. DDPG	2D Path search time	<0.001	0.952
	2D Path length	<0.001	≈0.99
SA-TD3-RRT vs. DQN	2D Path search time	<0.001	0.98
	2D Path length	<0.001	0.969
RRT	2D Path search time	0.032	0.843
	2D Path length	<0.001	≈0.99
Improved RRT	2D Path search time	<0.001	0.448
	2D Path length	0.182	0.68

**Table 6:** Comparative experimental results in a sparse-target environment.

Algorithm	2D Path Average Search Time (s)	2D Path Average Length (mm)	2D Path Nodes	3D Path Average Search Time (s)	3D Path Average Length (mm)	3D Post-Processed Nodes
TD3	4 ± 0.76	2142 ± 91.48	5496.7 ± 96.2	18.96 ± 0.07	14,140.07 ± 107.2	30,471
PPO	9.4 ± 0.02	5495.37 ± 44.74	4002.6 ± 1.9	3 ± 0.03	17,493.44 ± 169.64	30,471
SAC	3.1 ± 0.02	2109.17 ± 79.96	6545.6 ± 483.3	3 ± 0.02	14,107.25 ± 464.76	30,471
DDPG	3.2 ± 0.03	2024.09 ± 44.52	4001.9 ± 330.7	2.1 ± 0.03	14,022.16 ± 344.52	30,471
DQN	5 ± 0.01	8439.63 ± 90.54	3822.5 ± 42.4	2.3 ± 0.03	20,437.7 ± 190.84	30,471
SA-TD3-RRT	2.94 ± 0.11	796.08 ± 37.41	8	3.13 ± 0.1	12,947.15 ± 312.41	30,471

**Table 7:** Comparative experimental results in a multi-target environment.

Algorithm	2D Path Search Time (s)	2D Path Average Length (mm)	2D Path Nodes	3D Path Average Search Time (s)	3D Path Average Length (mm)	3D Post-Processed Nodes
TD3	8 ± 0.26	9977.06 ± 134.01	34,223.7 ± 6357.3	77.84 ± 1.03	78,932.54 ± 434.01	152,647
PPO	5.25 ± 0.15	33,604.91 ± 164.99	22,913 ± 472	11 ± 0.05	102,560.4 ± 243.16	152,647
SAC	1.61 ± 0.05	11,482.11 ± 124.95	22,118.7 ± 844.5	11.56 ± 0.05	83,437.0 ± 224.18	152,647
DDPG	1.65 ± 0.1	14,754.17 ± 246.2	22,340.1 ± 586.2	9.67 ± 0.08	83,709.66 ± 306.04	152,647
DQN	2.3 ± 0.05	49,088.45 ± 112.31	22,197.4 ± 472	10.4 ± 0.05	118,043.94 ± 1229.07	152,647
SA-TD3-RRT	1.74 ± 0.08	1930.03 ± 104.1	46	17.62 ± 0.06	70,885.51 ± 164.11	152,031

## Data Availability

The data that support the findings of this study are available within the manuscript.
